# Efficient synthesis of aziridinecyclooctanediol and 3-aminocyclooctanetriol

**DOI:** 10.3762/bjoc.18.163

**Published:** 2022-11-11

**Authors:** Emine Salamci, Ayse Kilic Lafzi

**Affiliations:** 1 Department of Chemistry, Faculty of Sciences, Atatürk University, 25240 Erzurum, Turkey

**Keywords:** aminocyclitols, aminocyclooctanetriol, azides, aziridines, aziridinecyclooctanediol

## Abstract

Cyclooctene endoperoxide was used as the key compound for the synthesis of aziridinecyclooctanediol and 3-aminocyclooctanetriol. Reduction of the cyclooctene endoperoxide, prepared by photooxygenation of *cis,cis*-1,3-cyclooctadiene, with zinc gave a cyclooctenediol and then benzylation of the hydroxy group yielded dibenzylated cyclooctene. Oxidation of the latter compound by OsO_4_/NMO followed by mesylation of the hydroxy group provided bis(benzyloxy)cyclooctane-1,2-diyl dimethanesulfonate. Reaction of the bis(benzyloxy)cyclooctane-1,2-diyl dimethanesulfonate with NaN_3_ gave 2-azido-3,8-bis(benzyloxy)cyclooctyl methanesulfonate. Reduction of the azide group and debenzylation to give an amine provided the new 3-aminocyclooctanetriol. Treatment of the 2-azido-3,8-bis(benzyloxy)cyclooctyl methanesulfonate with Zn/NH_4_Cl and debenzylation resulted in the target aziridinecyclooctanediol.

## Introduction

**Figure 1:**
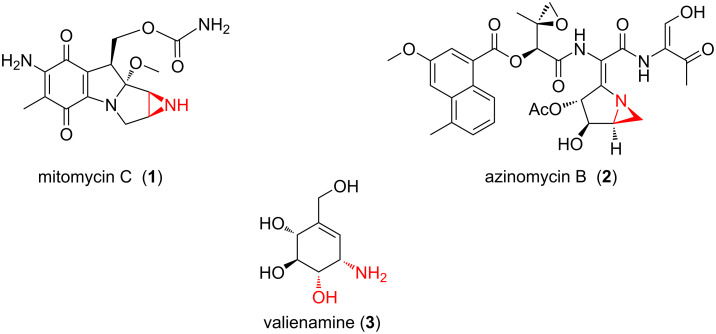
Some biologically active aziridine-bearing compounds **1**, **2** and aminocyclitol **3**.

Aziridines are the smallest nitrogen-containing heterocycles and they are important building blocks in the synthesis as well as substructures of a number of biologically active natural and unnatural products [1–8]. Aziridines are valuable synthetic intermediates for the preparation of structurally complex molecules because of their versatility in numerous regio- and stereoselective ring opening and/or expansion reactions, as well as rearrangements [5]. The aziridine structural motif is present in natural products such as mitomycins and azinomycins (Figure 1) [1,5], which exhibit potent biological activities such as antitumor and antibiotic activities.

Therefore, synthetic methodologies for the preparation of the aziridinyl system have attracted attention in recent decades. Opening of the aziridine ring by using different nucleophiles gives the corresponding amino alcohols, amino esters, azido amines, amines, and other derivatives [9]. Furthermore, aziridine derivatives are valuable precursors for the synthesis of aminocyclitols, which can be found in nature in several families of natural and clinically important antibiotics [10].

Aminocyclitols containing the amino alcohol motif are important structural components for modifying bioactive natural products and pharmaceuticals. Valienamine (**3**) and its analogues show inhibitory activity against certain glycosidases [11–13] (Figure 1).

Many groups have described different synthetic methods for the synthesis of various aminocyclitols [13–17]. However, only few synthetic methods are available for the synthesis of eight-membered [18–29] aminocyclitols. On the other hand, the synthesis of a C8-cyclitol derivative containing the aziridine ring has not yet been reported. Therefore, in our continued efforts for efficient syntheses of cyclitols [30–33] and C8-aminocyclitols [18–24], we were interested in developing an efficient synthesis of aziridinecyclooctanediol. In the present paper, we report the efficient synthesis of aziridinecyclooctanediol and a new 3-aminocyclooctanetriol stereoisomer starting from *cis,cis*-1,3-cyclooctadiene.

## Results and Discussion

**Scheme 1:**
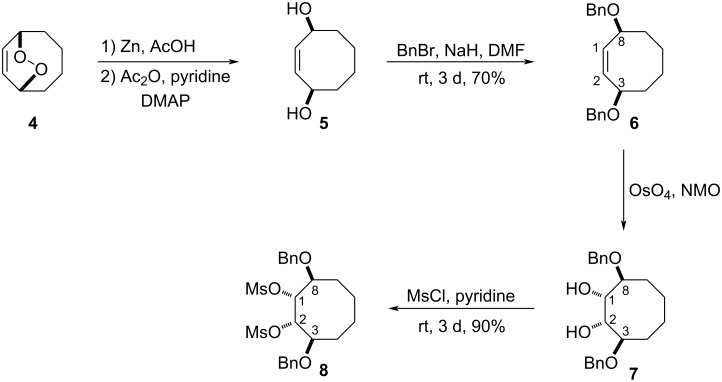
Synthesis of dimesylate **8**.

The synthesis of the diol **5**, which was prepared by reduction of the endoperoxide **4** with zinc was carried out as described in the literature [18]. Treatment of the diol **5** with benzyl bromide and NaH in DMF gave the corresponding (dibenzyloxy)cyclooctene **6** in 70% yield (Scheme 1). Oxidation of the dibenzylated compound **6** with OsO_4_/NMO provided the corresponding diol **7** in 90% yield. The exact configuration of **7** was confirmed by ^1^H and 2D NMR spectroscopic data. Next, mesylation of the hydroxy groups in **7** with MsCl in pyridine yielded dimesylate **8** in 90% yield. Thus, the dimesylate **8**, which is one of the most relevant precursors for the synthesis of aminocyclitols, was synthesized from the diol **7**. The structure of compound **8** was assigned on the basis of NMR spectroscopy. In the ^1^H NMR spectrum, we observed that methyl signals of the mesylate groups in **8** gave a multiplet, although compound **8** is symmetrical. To determine the existence of a dynamic process in the molecule **8**, the NMR spectra of **8** were recorded at different temperatures. With an increase in the temperature, only one signal for the mesylate groups was observed in the spectrum. This difference is due to the occurrence of dynamic balance when the system is heated.

**Scheme 2:**
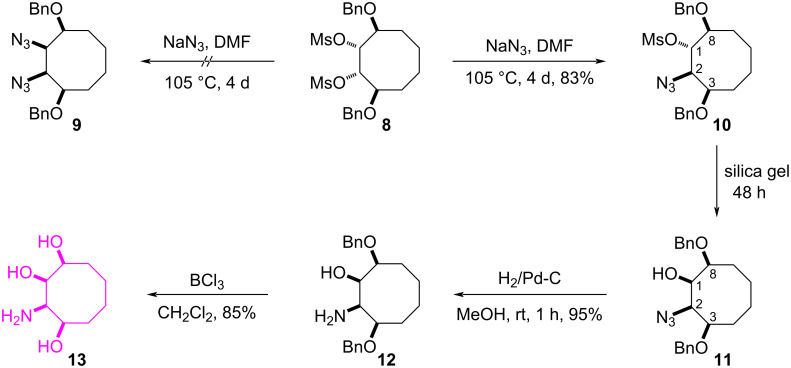
Synthesis of aminocyclooctanetriol **13**.

A general and versatile method for the synthesis of azides involves the reaction of a mesylate with sodium azide. Therefore, the dimesylate **8** was first reacted with an excess of sodium azide in DMF at 105 °C to give the diazide **9** (Scheme 2). However, the product was determined to be the azidomesylate **10** instead of the expected diazide **9** based on NMR spectroscopy. The position of the azide functionality in **10** was determined from its COSY spectrum. The diagonal peak at 3.97 ppm has cross peaks with the protons resonating at 3.84 and 4.92 ppm. Analysis of these cross peaks shows that the cross peak at 4.92 ppm is strong. This strong correlation supports the *trans* relation of the protons H-2 and H-1.

Next, in the preparation of the azidomesylate **10** from dimesylate **8**, when the amount of **8** was increased (from milligrams to grams), the azido alcohol **11** was obtained during purification of the crude product from DMF. When the crude product **10** remains on the silica gel column with EtOAc/*n*-hexane 2:8 followed by methanol as the eluent for 48 hours to remove DMF, we determined from the NMR spectra that the mesylate group in compound **10** was converted to the corresponding alcohol **11** as the sole isomer via S_N_2 substitution by hydrolysis. In the azidolysis reaction of compound **8**, we propose that because the azide group is bulkier than the water molecule, it could not substitute the second mesylate group, and therefore the diazide **9** could not form. The configuration of the hydroxy group in **11** was determined by the cross peak between the proton H-2 and the protons H-1 and H-3 in the COSY spectrum. Moreover, the fact that the proton H-1 gives a positive NOE clearly indicates that it should have a *cis* configuration relative to the proton H-2. For the synthesis of the aminocyclooctanetriol **13**, hydrogenation of the azido alcohol **11** gave amine **12** in 95% yield (Scheme 2). Subsequent, benzyl deprotection with BCl_3_ of **12** resulted in the target compound **13** in 85% yield. The structures of compounds **12** and **13** are completely in agreement with our NMR spectral findings.

**Scheme 3:**
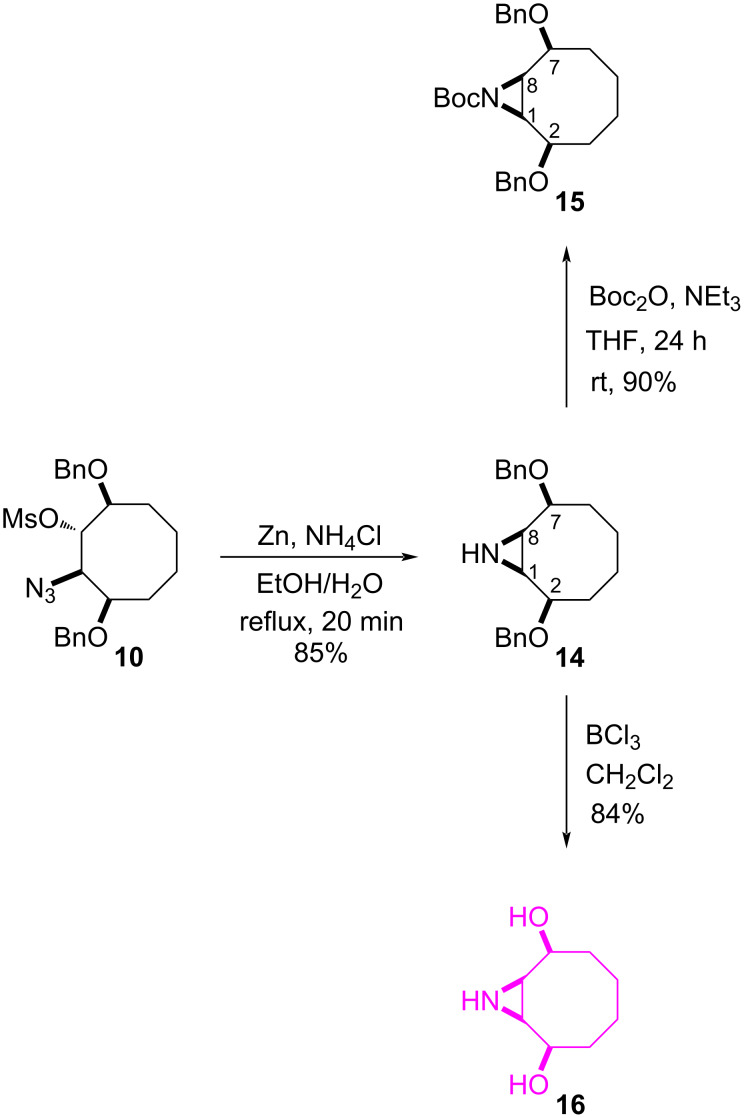
Synthesis of aziridinecyclooctanediol **16**.

We then turned our attention to the synthesis of an aziridine-fused cyclooctane derivative from azidomesylate **10**. Treatment [34] of **10** with Zn powder in the presence of NH_4_Cl in EtOH/H_2_O resulted in the corresponding aziridine **14** as a single product (Scheme 3). For further structural proof, the aziridine **14** was converted into the corresponding *N*-Boc-protected aziridine ester **15** with Boc_2_O/NEt_3_ in THF (yield 90%). Again, the structure of **15** was confirmed by 1D (^1^H and ^13^C) and 2D (COSY, NOE, and HMQC) NMR spectroscopic data. The protons H-2/H-7 giving a positive NOE clearly indicates that the protons H-2/H-7 should have a *cis* configuration relative to the protons H-1/H-8. Finally, benzyl deprotection with BCl_3_ of **14** afforded the product **16** in 84% yield. The structure of **16** was assigned on the basis of NMR spectroscopy.

## Conclusion

In summary, we have achieved the synthesis of 3-aminocyclooctanetriol **13** and aziridinecyclooctanediol **16** starting from *cis,cis*-1,3-cyclooctadiene. The nitrogen functionalities were introduced by the substitution with NaN_3_ of the corresponding mesylate. Reduction of the azido functionalities gave monoaminocyclitol and aziridine-fused derivatives.

## Supporting Information

File 1 Experimental section, ^1^H and ^13^C NMR spectra for all new compounds, as well as selected 2D NMR spectra are provided.
