# Supplementary material for: Efficient synthesis of aziridinecyclooctanediol and 3-aminocyclooctanetriol
Source: Beilstein J Org Chem. 2022 Nov 11;18:1539–43. doi: 10.3762/bjoc.18.163 (PMC9663974; doi:10.3762/bjoc.18.163)

BEILSTEIN JOURNAL OF ORGANIC CHEMISTRY

Supporting Information

for

Efficient synthesis of aziridinecyclooctanediol and 3-aminocyclooctanetriol

Emine Salamci and Ayse Kilic Lafzi

Beilstein J. Org. Chem. **2022,** *18,* 1539–1543. doi:10.3762/bjoc.18.163

Experimental section, ^{1}H and ^{13}C NMR spectra for all new compounds, as well as selected 2D NMR spectra are provided

License and Terms: This is a supporting information file under the terms of the Creative Commons Attribution License (https://creativecommons.org/licenses/by/4.0). Please note that the reuse, redistribution and reproduction in particular requires that the author(s) and source are credited and that individual graphics may be subject to special legal provisions.
The license is subject to the *Beilstein Journal of Organic Chemistry* terms and conditions: (https://www.beilstein-journals.org/bjoc/terms)

Table of contents

Experimental section S1
General information S1
Syntheses S1
References S4
Copies of spectra S5

Experimental section

General information

Melting points are uncorrected. Infrared spectra were obtained from solution in 0.1 mm cells or KBr pellets on an FT-IR Mattson 1000 instrument. The ^{1}H and ^{13}C NMR spectra were recorded on 400 (100) MHz Varian or 400 (100) MHz Bruker spectrometer and are reported in δ units with $SiMe_4$ as internal standard. HRMS spectra were obtained on a Bruker microTOF-Q or Agilent 6530 Accurate Mass Q-TOF instrument. Melting points were determined on a GallenKamp MPD 350. Column chromatography was performed on silica gel (60 mesh, Merck). TLC was carried out on Merck 0.2 mm silica gel 60 F_{254} analytical aluminium plates.

Syntheses

7,8-Dioxabicyclo[4.2.2]dec-9-ene (4): Compound **4** was prepared as described in the literature [1].

***cis*-2-Cyclooctene-1,4-diol (5):** Compound **5** was prepared as described in the literature [1].

(3*R,8*S**,*Z*)-3,8-Bis(benzyloxy)cyclooct-1-ene (6):** To a magnetically stirred solution of diol **5** (500 mg, 3.52 mmol) in absolute DMF (10 mL) at 0 °C and NaH (422 mg, 17.58 mmol) was added. After stirring the mixture at the same temperature for 45 min benzyl bromide (2.71 g, 15.82 mmol) was added to the flask. The mixture was stirred at room temperature for 3 d. After the reaction was completed (monitored by TLC), ether (10 mL) was added and stirred for 10 min. The reaction mixture was quenched with water (12 mL) and then extracted with ether (5 × 20 mL). The combined organic layers were washed with water (2 × 10 mL), dried (Na_2SO_4), and concentrated under reduced pressure. The solvent was removed under reduced pressure, and the crude product was purified by chromatography on a silica gel column (70 g) eluting with ether/hexane 10:90 to give pure dibenzylated compound **6** (795 mg, 70%), as a colourless oil. ^{1}H-NMR (400 MHz, $CDCl_3$): δ 7.44-7.30 (m, 10H, Ph), 5.81-5.73 (m, 2H, H-1 and H-2), 4.67 (d, J = 11.9 Hz, 2H, OCH_AH_BPh, A part of AB system), 4.46 (d, J = 11.9 Hz, 2H, OCH_AH_BPh, B part of AB system), 4.22-4.11 (m, 2H, H-3 and H-8), 2.11-1.90 (m, 2H, CH_2), 1.66-1.41 (series of m, 6H, CH_2). ^{13}C-NMR (100 MHz, $CDCl_3$): δ 138.7, 134.3, 128.7, 128.1, 127.9, 75.8, 70.9, 36.3, 24.0. IR (KBr, cm^{-1}): 3029, 2930, 2859, 1743, 1496, 1453, 1382, 1362, 1300, 1227,1147, 1070, 1029, 931, 858. HRMS (ESI-TOF) m/z: $[M + H]^+$ calcd for $C_{22}H_{26}O_2$: 323.2011; found: 323.2006.

Oxidation of dibenzylated compound 6 with OsO_4/NMO: In a similar manner as described in the literature [1] to a stirred solution of dibenzylated compound **6** (3.61 g, 11.19 mmol) in acetone (26 mL) were added a solution of NMO (2.26 g, 16.74 mmol) in 21 mL of water and OsO_4 (ca. 427 mg, 1.68 mmol) and cooled to 0 °C. The resulting mixture was stirred vigorously under nitrogen at room temperature and after 8 d, the reaction was complete. The reaction was quenched with $NaHSO_3$ (29.72 g) and florisil (9.30 g) solution in water (82 mL) and stirred for 10 min, and then filtered through a pad of Celite (29.72 g) in a 250 mL sintered glass funnel. The Celite cake was washed with acetone (4 × 40 mL). The filtrate was neutralized to pH 7 with H_2SO_4 (12 N). The organic layer was removed in vacuo. The pH of the resulting aqueous solution was adjusted to pH 5, and extracted with ethyl acetate (5 × 30 mL). The combined organic layers were washed with 30 mL of 25% NaCl solution and dried over Na_2SO_4. Evaporation of solvent gave pure diol **7** (3.60 g, 90%) as the sole product. The diol **7** was recrystallized from EtOAc/hexane as a white solid; mp 108-110 °C. (1*R**,2*S**,3*S**,8*R**)-3,8-bis(benzyloxy)cyclooctane-1,2-diol (**7**): ^{1}H-NMR (400 MHz, $CDCl_3$): δ 7.39-7.25 (m, 10H, Ph), 4.65 (d, J = 11.6 Hz, 2H, OCH_AH_BPh, A part of AB system), 4.50 (d, J = 11.6 Hz, 2H, OCH_AH_BPh, B part of AB system), 4.02-3.97 (m, 2H, H-1 and H-2), 3.69-3.62 (m, 2H, H-3 and H-8), 2.82-2.78 (bs, 2H, OH), 1.98-1.45 (series of m, 8H, CH_2). ^{13}C-NMR (100 MHz, $CDCl_3$): δ 138.5, 128.5, 127.7, 80.4, 74.3, 71.2, 28.4, 24.6. IR (KBr,

cm^{-1}): 3417, 3380, 3029, 2915, 2896, 1736, 1453, 1341, 1210, 1158, 1056, 1012, 980, 886. HRMS (APCI-TOF) m/z: $[M + H]^+$ calcd for $C_{22}H_{28}O_4$: 357.2066; found: 357.2057.

Synthesis of (1*R,2*S**,3*R**,8*S**)-3,8-bis(benzyloxy)cyclooctane-1,2-diyl dimethanesulfonate (8):** In a similar manner as described in the literature [2] to a magnetically stirred solution of diol **7** (500 mg, 1.40 mmol) in pyridine (4.0 mL) cooled to 0 °C, MsCl (0.65 mL, 8.38 mmol) was added and the mixture was stirred at room temperature for 72 h. Then, the mixture was cooled to 0 °C and 180 mL of 1 M HCl solution was added, and the mixture was extracted with CH_2Cl_2 (6 × 20 mL). The combined organic extracts were washed with saturated $NaHCO_3$ solution (70 mL) and water (3 × 8 mL) and then dried over Na_2SO_4. Evaporation of the solvents gave pure **8** (645 mg, 90%); recrystallized from CH_2Cl_2/hexane as a white solid; mp 139-140 °C. ^{1}H-NMR (400 MHz, $CDCl_3$): δ 7.39-7.24 (m, 10H, Ph), 5.22-5.16 (m, 2H, H-1 and H-2), 4.65-4.52 (m, 4H, OCH_2Ph), 3.88-3.81 (m, 2H, H-3 and H-8), 2.99 (s, 6H, OCH_3), 2.10-1.45 (series of m, 8H, CH_2). ^{13}C-NMR (100 MHz, $CDCl_3$): δ 137.8, 128.7, 128.5, 128.4, 128.2, 128.1, 85.6, 82.9, 79.4, 77.6, 71.6, 38.8, 27.2, 24.8, 23.3, 20.1. IR (KBr, cm^{-1}): 3063, 3032, 2938, 2872, 2309, 1959, 1734, 1605, 1497, 1454, 1356, 1178, 1070, 1028, 970, 920, 844. HRMS (ESI-TOF) m/z: $[M + H]^+$ calcd for $C_{24}H_{32}O_8S_2$: 513.1617; found: 513.1605.

Reaction of (1*R,2*S**,3*R**,8*S**)-3,8-bis(benzyloxy)cyclooctane-1,2-diyl dimethanesulfonate (8) with NaN_3**

a) To a stirred solution of dimesylate **8** (0.5 g, 0.97 mmol) in DMF (6 mL) was added NaN_3 (1.58 g, 24.30 mmol), followed by stirring at 105 °C for 4 d. After the reaction was completed (monitored by TLC), water (15 mL) was added and the mixture was allowed to stir for 30 min, followed by extraction with ethyl acetate (4 × 20 mL). The combined organic extracts were washed with water (4 × 10 mL) and then dried over Na_2SO_4. The solvent was removed under reduced pressure and the crude product was purified by column chromatography eluting with EtOAc/*n*-hexane 2:8 to give monoazide **10** as a colourless oil (370 mg, 83%). (1*S**,2*R**,3*R**,8*S**)-2-azido-3,8-bis(benzyloxy)cyclooctyl methanesulfonate (**10**) ^{1}H-NMR (400 MHz, $CDCl_3$): δ 7.42-7.28 (m, 10H, Ph), 4.92 (t, *J* = 8.0 Hz, 1H, H-1), 4.71-4.53 (m, 4H, OCH_2Ph), 3.97 (dd, *J* = 7.6 Hz, *J* = 1.9 Hz, 1H, H-2), 3.90-3.75 (m, 1H, H-3), 3.66-3.59 (m, 1H, H-8), 2.98 (s, 3H, OCH_3), 2.15-1.36 (series of m, 8H, CH_2). ^{13}C-NMR (100 MHz, $CDCl_3$): δ 137.8, 137.7, 128.5, 128.4, 127.9, 127.8, 127.6, 83.2, 78.0, 77.6, 72.0, 71.2, 65.9, 38.7, 28.2, 27.5, 22.3, 22.1. IR (KBr, cm^{-1}): 3030, 2930, 2857, 2103, 1717, 1453, 1353, 1260, 1172, 1069, 941, 836. HRMS (APCI-TOF) m/z: $[M - N_2 + H]^+$ calcd for $C_{23}H_{29}NO_5S$: 432.1845; found: 432.1842.

b) After the same procedure as described above for compound **10** was applied to the dimesylate **8** (1.0 g, 1.95 mmol) in DMF (12 mL), during purification of the crude product from DMF by column chromatography, eluting with EtOAc/*n*-hexane 2:8 and after 48 hours eluting with MeOH on silica gel column gave pure azido alcohol **11** as a colourless oil (640 mg, 86%).

(1*R,2*S**,3*R**,8*S**)-2-Azido-3,8-bis(benzyloxy)cyclooctan-1-ol (11)** ^{1}H-NMR (400 MHz, $CDCl_3$): δ 7.48-7.27 (m, 10H, Ph), 4.68 (d, *J* = 11.9 Hz, 1H, OCH_AH_BPh, A part of AB system), 4.64 (d, *J* = 11.9 Hz, 1H, OCH_AH_BPh, B part of AB system), 4.58-4.49 (m, 2H, OCH_2Ph), 4.28-4.19 (m, 1H, H-8), 3.99-3.94 (m, 1H, H-1), 3.59-3.51 (m, 1H, H-3), 3.50-3.43 (m, 1H, H-2), 2.28-1.49 (series of m, 8H, CH_2). ^{13}C-NMR (100 MHz, $CDCl_3$): δ 138.4, 138.3, 128.5, 128.4, 127.7, 127.6, 75.7, 75.0, 71.2, 70.4, 65.9, 61.3, 36.9, 33.2, 30.5, 26.9. IR (KBr, cm^{-1}): 3394, 3189, 2921, 2849, 2096, 1646, 1454, 1272, 1068, 736, 698. HRMS (APCI-TOF) m/z: $[M - N_2 + H]^+$ calcd for $C_{22}H_{27}NO_3$: 354.2069; found: 354.2070.

(1*R,2*S**,3*R**,8*S**)-2-Amino-3,8-bis(benzyloxy)cyclooctan-1-ol (12):** In a similar manner as described in the literature [3] in a 50 mL flask was placed palladium on charcoal (20 mg, 10%) and azidol **11** (140 mg, 0.367 mmol) in absolute methanol (15 mL). The reaction mixture was flushed with hydrogen gas (the air in the solvent was removed under vacuum, and then the flask was filled with hydrogen gas; this process was repeated three times). The resulting mixture was stirred at room temperature for 1 h under the hydrogen atmosphere. The catalyst was removed by filtration. Evaporation of the solvent gave pure aminotriol **12** (124 mg, 95%) as a colourless oil. ^{1}H-NMR (400 MHz, $CDCl_3$): δ 7.41-7.23 (m, 10H, Ph), 4.62 (d, *J* = 11.8 Hz, 1H, OCH_AH_BPh, A part of AB system), 4.49 (d, *J* = 11.8 Hz, 1H, OCH_AH_BPh, B part of AB system), 4.49 (s, OCH_2Ph, 2H), 4.26-4.16 (m, H-8, 1H), 3.82-3.74 (m, H-1, 1H), 3.58-3.48 (m, H-3, 1H), 3.05 (brd, *J* = 10.4 Hz, H-2, 1H), 2.30-1.50 (series of m, 11H, NH_2, OH and CH_2). ^{13}C-NMR (100 MHz, $CDCl_3$): δ 138.8, 128.4, 127.6, 127.5, 76.2, 75.5, 70.8, 70.2, 66.4, 50.7, 37.0, 35.8, 30.3, 27.0. IR (KBr, cm^{-1}): 3353, 3064, 3030, 2920, 2851, 2100, 1734, 1586, 1496, 1453, 1361, 1309, 1202, 1065, 1027. HRMS (ESI-TOF) m/z: $[M + H]^+$ calcd for $C_{22}H_{29}NO_3$: 356.2226; found: 356.2223.

(1*S,2*R**,3*R**,4*R**)-3-Aminocyclooctane-1,2,4-triol (13):** In a similar manner as described in the literature [3] to a stirred solution of bis(benzyloxy)aminoalcohol **12** (110 mg, 0.3 mmol) in CH_2Cl_2 (21 mL) cooled to −78 °C, was added BCl_3 (1.386 mL, 1.55 mmol, 1 M in hexane). After the mixture was stirred for 2 h at this temperature, it was warmed to 0 °C gradually and stirred for 10 h. Then, the reaction was quenched with MeOH (7 mL) at −78 °C and stirred for 1 h. Evaporation of the solvents gave aminotriol **13** as a colourless oil (45 mg, 83%). ^{1}H-NMR (400 MHz, CD_3OD): δ 4.22-4.14 (m, 1H, H-2), 4.12-4.03 (m, 1H, H-1), 3.90-3.80 (m, 1H, H-4), 3.43-3.37 (m, 1H, H-3), 2.20-1.22 (series of m, 8H, CH_2). ^{13}C-NMR (100 MHz, CD_3OD): δ 69.2, 65.9, 64.8, 51.1, 38.1, 33.8, 30.3, 29.3. IR (KBr, cm^{-1}): 3359, 3042, 2933, 2106, 1619, 1470, 1409, 1272, 1015, 908. HRMS (ESI-TOF) m/z: $[M + H]^+$ calcd for $C_8H_{17}NO_3$: 176.1287; found: 176.1278.

Reaction with Zn and NH_4Cl of azidomesylate 10: In a similar manner as described in the literature [4], to a solution of azidomesylate **10** (300 mg, 0.65 mmol) and NH_4Cl (810 mg, 1.51 mmol) in EtOH/H_2O 3:1 (10 mL), zinc powder (560 mg, 0.86 mmol) was added and the mixture was stirred vigorously at reflux temperature for 20 min. After the reaction was completed (monitored by TLC), ethyl acetate (100 mL) was added. Then, the mixture was filtered, and the filtrate was washed with brine and dried over Na_2SO_4. Evaporation of the solvent gave pure dibenzylaziridine **14** as a colourless oil (188 mg, 85%). (1*R**,2*S**,7*R**,8*S**)-2,7-bis(benzyloxy)-9-azabicyclo[6.1.0]nonane **(14)** ^{1}H-NMR (400 MHz, $CDCl_3$): δ 7.80-7.20 (m, 10H, Ph), 4.65 (d, *J* = 12.0 Hz, 2H, OCH_AH_BPh, A part of AB system), 4.57 (d, *J* = 12.0 Hz, 2H, OCH_AH_BPh, B part of AB system), 3.88-3.78 (m, 2H, H-2 and H-7), 2.43 (brs, 2H, H-1 and H-8), 2.10-1.10 (series of m, 8H, CH_2). ^{13}C-NMR (100 MHz, $CDCl_3$): δ 138.9, 128.6, 127.8, 127.7, 76.7, 70.9, 38.9, 25.6, 23.3. IR (KBr, cm^{-1}): 3304, 3029, 2924, 2854, 1735, 1495, 1454, 1377, 1205, 1088, 1066, 1028, 877. HRMS (APCI-TOF) m/z: $[M + H]^+$ calcd for $C_{22}H_{27}NO_2$: 338.2120; found: 338.2122.

***tert*-Butyl (1*R**,2*S**,7*R**,8*S**)-2,7-bis(benzyloxy)-9-azabicyclo[6.1.0]nonane-9-carboxylate (15):** To a stirred solution of aziridine **14** (140 mg, 0.415 mmol) in absolute THF (7 mL) at 0 °C was added Et_3N (0.069 mL, 0.494 mmol) and Boc_2O (100 mg, 0.458 mmol) under a dry nitrogen atmosphere. After the mixture was stirred for 1 h at this temperature the solution was warmed to room temperature and stirred for 24 h. The reaction was quenched with saturated solution of NH_4Cl (25 mL) and extracted with EtOAc (100 mL). The combined organic phase was dried over Na_2SO_4 and the solvent was evaporated to afford pure compound **15** as a colourless oil (164 mg, 90%). ^{1}H-NMR (400 MHz, $CDCl_3$): δ 7.42-7.23 (m, 10H, Ph), 4.76 (d, *J* = 12.4 Hz, 2H, OCH_AH_BPh, A part of AB system), 4.72 (d, *J* = 12.4 Hz, 2H, OCH_AH_BPh, B part of AB system), 3.79-3.74 (m, 2H, H-2 and H-7), 2.78 (brs, 2H, H-1 and H-8), 1.47 (s, CH_3,

9H), 2.18-1.15 (series of m, 8H, CH_2). ^{13}C-NMR (100 MHz, $CDCl_3$): δ 163.3, 139.2, 128.5, 127.7, 127.5, 81.5, 75.9, 70.4, 44.5, 28.2, 26.4, 23.2. IR (KBr, cm^{-1}): 3327, 2917, 2849, 1717,1597, 1454, 1365, 1247, 1158, 1068. HRMS (APCI-TOF) m/z: $[M + H]^+$ calcd for $C_{27}H_{35}NO_4$: 438.2644; found: 438.2644.

(1*R,2*S**,7*R**,8*S**)-9-Azabicyclo[6.1.0]nonane-2,7-diol (16):** The aziridine **14** (120 mg, 0.356 mmol) was submitted to the debenzylation with BCl_3 following the method described above for the debenzylation of **12** to give diol **16**: 47 mg, 84%, as a colourless oil. ^1H-NMR (400 MHz, D_2O): δ 4.58-4.53 (m, 2H, H-2 and H-7), 3.32-3.28 (m, 2H, H-1 and H-8), 1.94-1.36 (series of m, 8H, CH_2). ^{13}C-NMR (100 MHz, D_2O): δ 62.7, 41.8, 33.2, 21.0. IR (KBr, cm^{-1}): 3221, 2923, 1594, 1462, 1110, 1051, 881. HRMS (APCI-TOF) m/z: $[M + H]^+$ calcd for $C_8H_{15}NO_2$: 158.1181; found: 158.1175.

References

1. Salamci, E. *Tetrahedron* **2010**, 66, 4010-4015.
2. Zozik, Y.; Salamci, E.; Kilic, A. *Tetrahedron Lett*. **2017**, 58, 4822-4826.
3. Karavaizoglu, U. N.; Salamci, E. *New J. Chem*. **2020**, 44, 17976-17983.
4. Lin, W.; Zhang, X.; He, Z.; Jin, Y.; Gong, L.; Mi, A. *Synth*. *Commun*. **2002**, 32, 3279-3284.

Copies of NMR spectra

(3*R**,8*S**,*Z*)-3,8-Bis(benzyloxy)cyclooct-1-ene (6): $CDCl_3$ (^{1}H NMR and ^{13}C NMR)

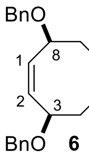

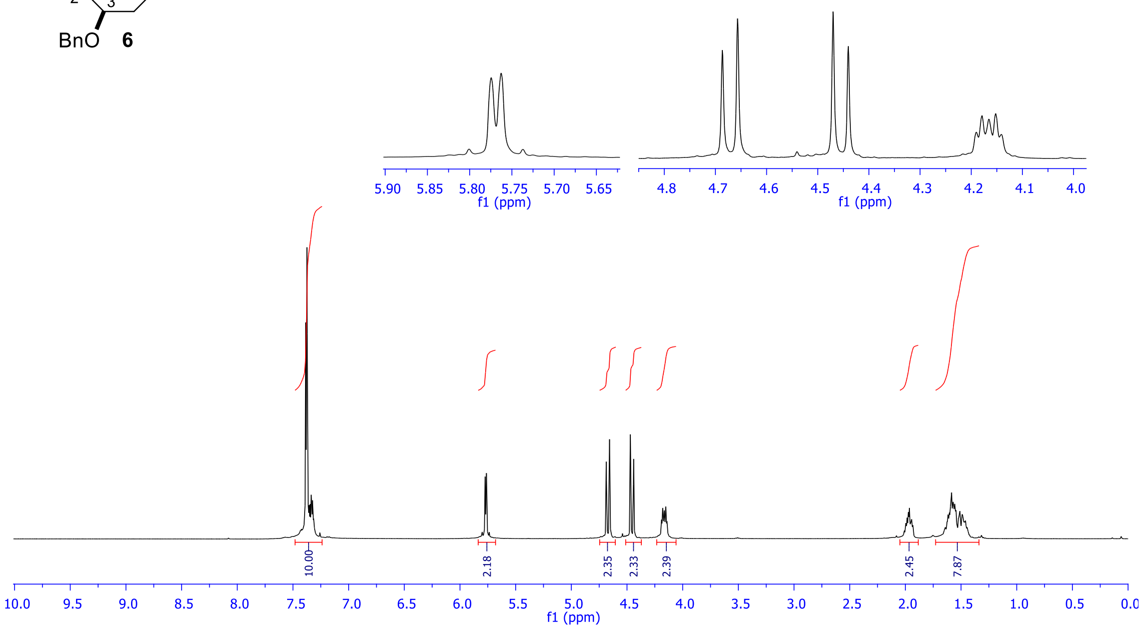

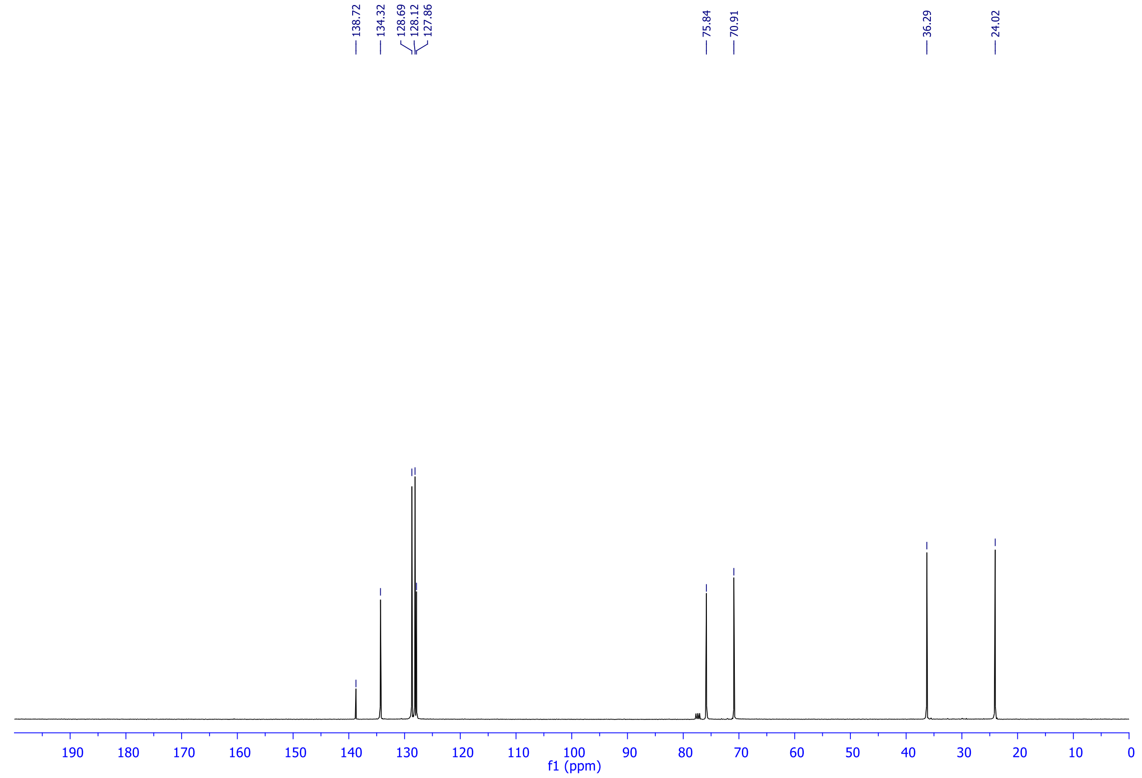

(1*R,2*S**,3*S**,8*R**)-3,8-Bis(benzyloxy)cyclooctane-1,2-diol (7): $CDCl_3$ (^{1}H NMR and ^{13}C NMR)**

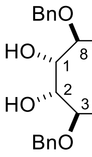

7

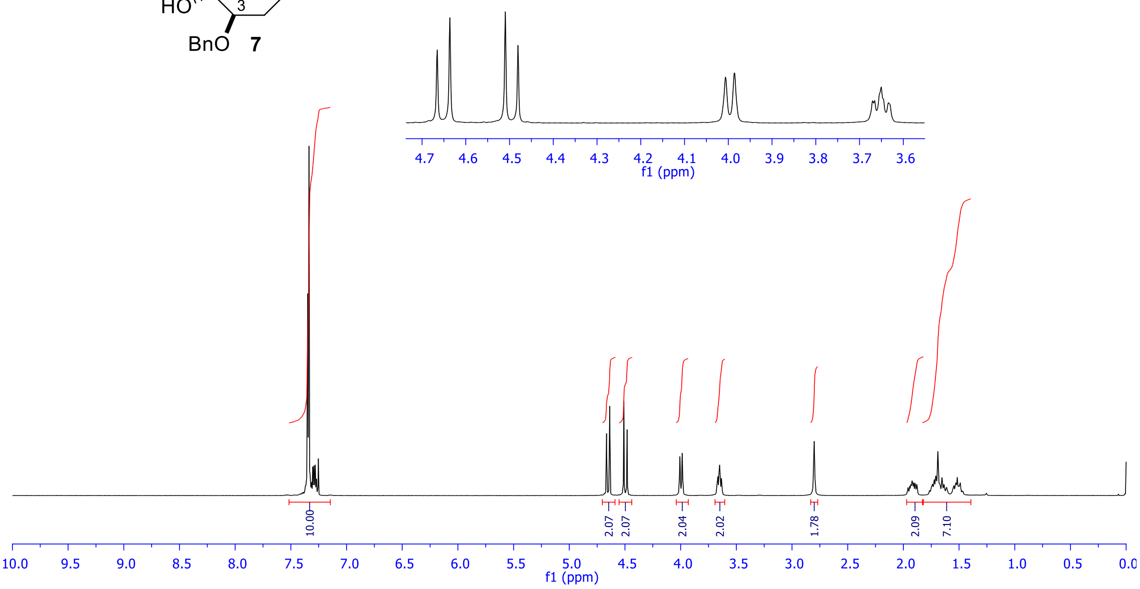

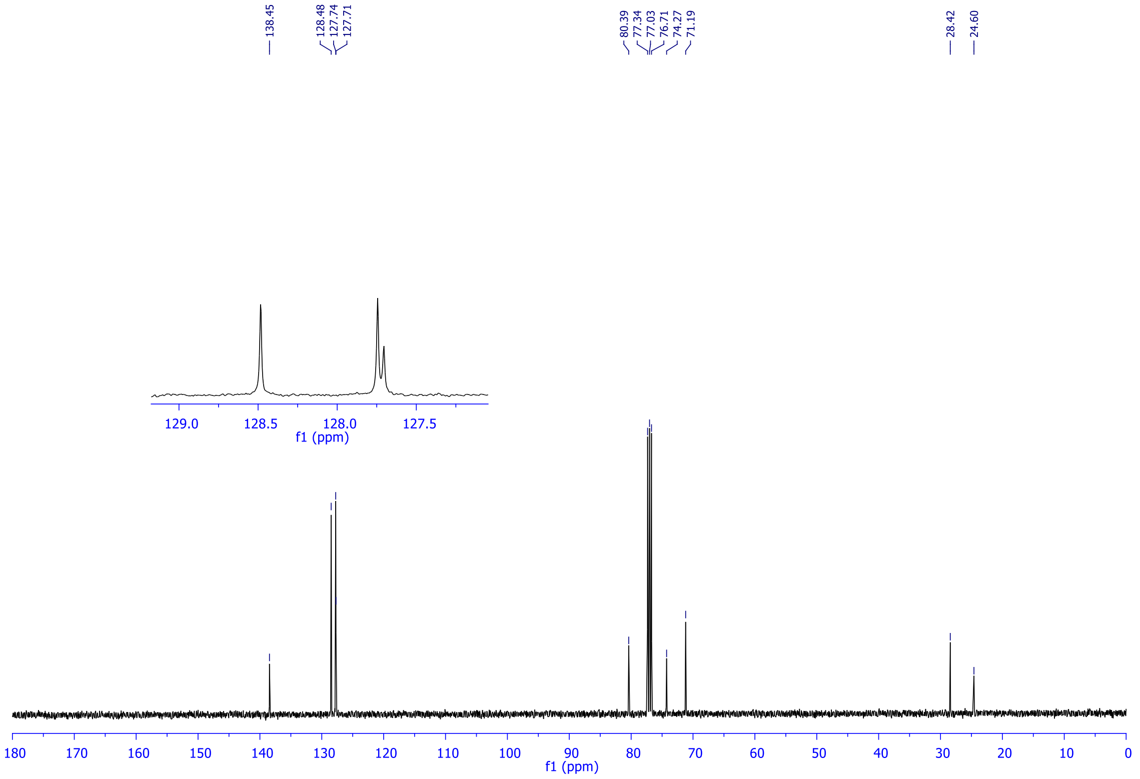

(1*R,2*S**,3*S**,8*R**)-3,8-Bis(benzyloxy)cyclooctane-1,2-diol (7): CDCl3-HMQC**

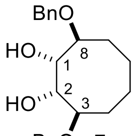

7

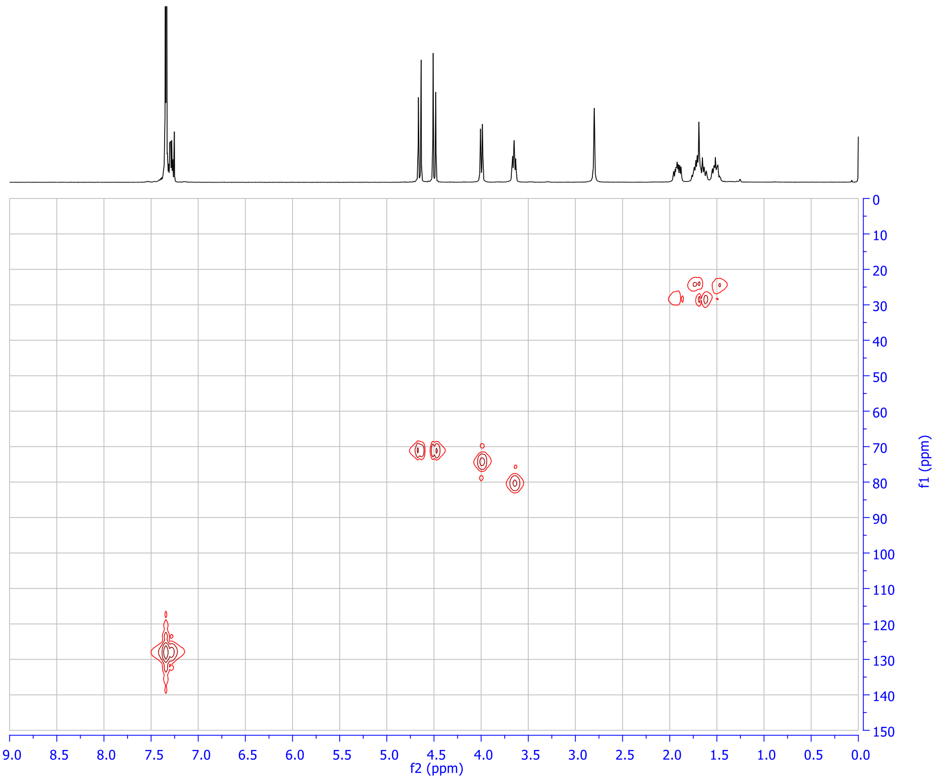

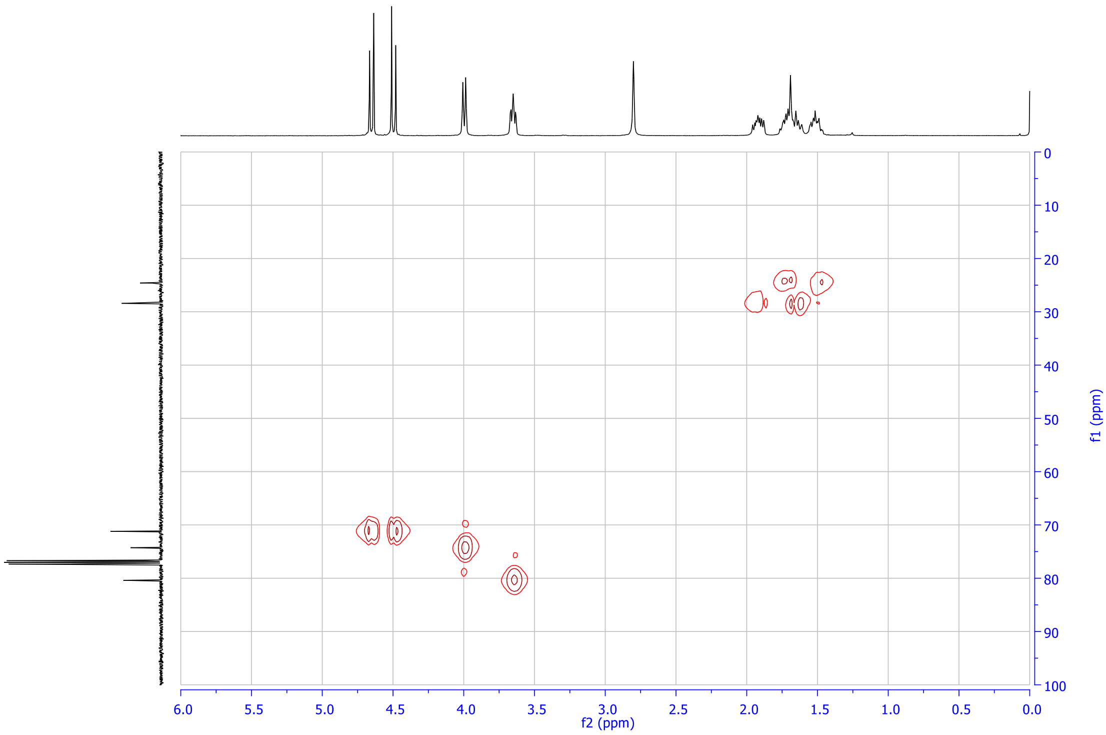

(1*R**,2*S**,3*S**,8*R**)-3,8-Bis(benzyloxy)cyclooctane-1,2-diol (7): $CDCl_3$-COSY

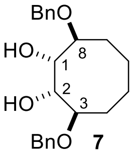

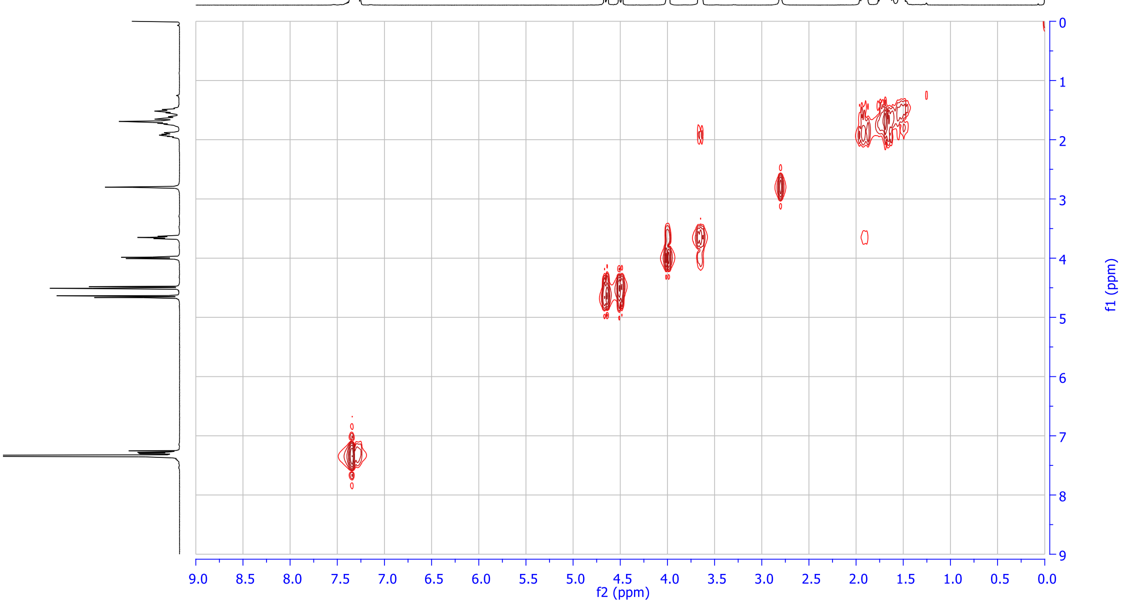

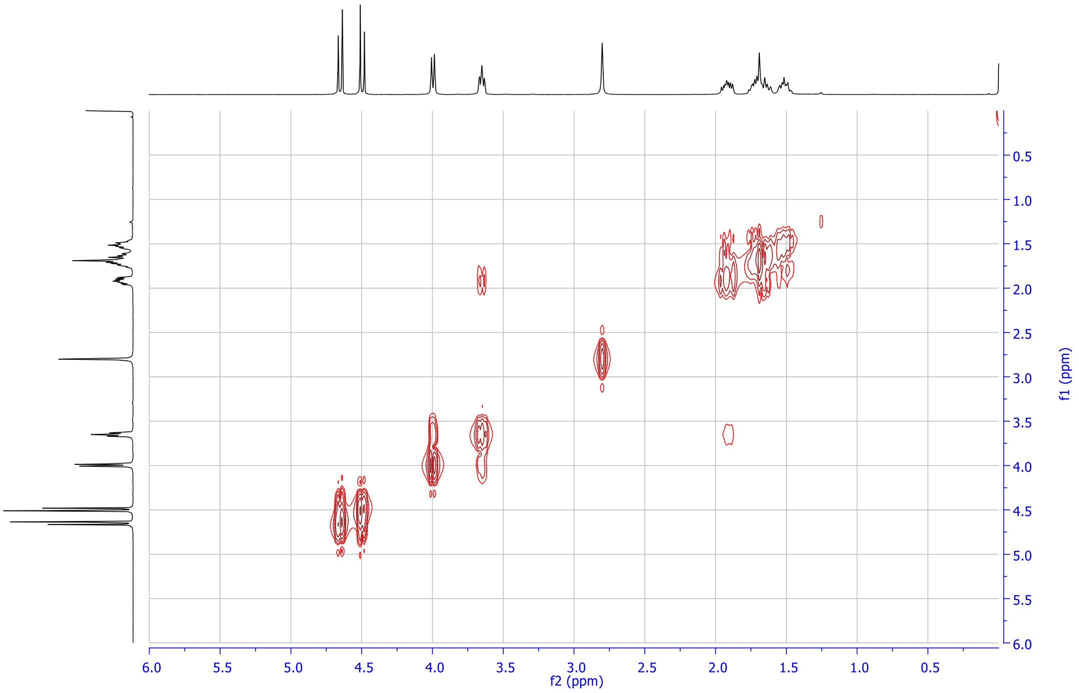

(1*R,2*S**,3*R**,8*S**)-3,8-Bis(benzyloxy)cyclooctane-1,2-diyl dimethanesulfonate (8): $CDCl_3$ (^{1}H NMR and ^{13}C NMR)**

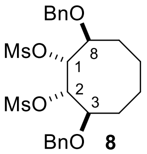

8

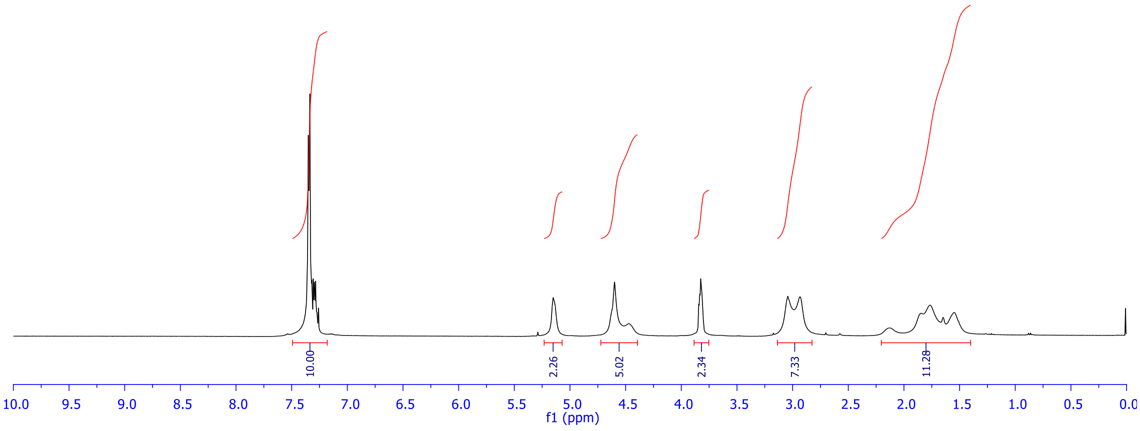

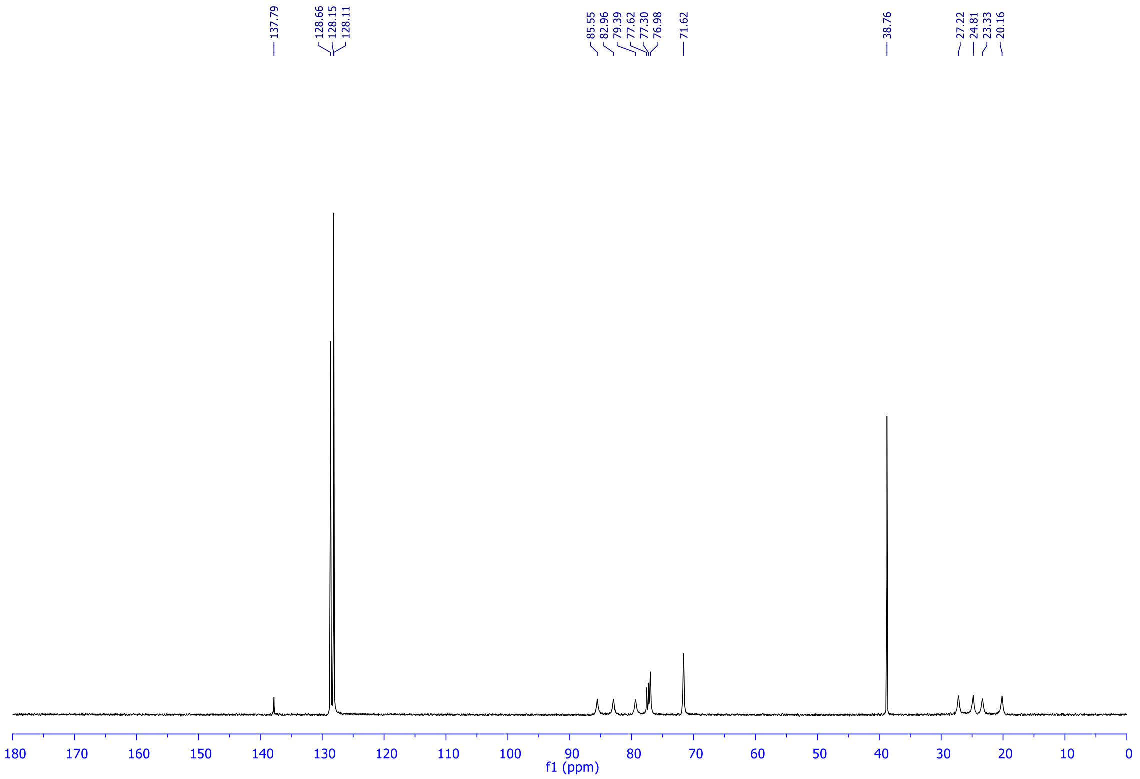

(1*R,2*S**,3*R**,8*S**)-3,8-Bis(benzyloxy)cyclooctane-1,2-diyl dimethanesulfonate (8): $CDCl_3$ (^1H NMR spectra at different temperatures)**

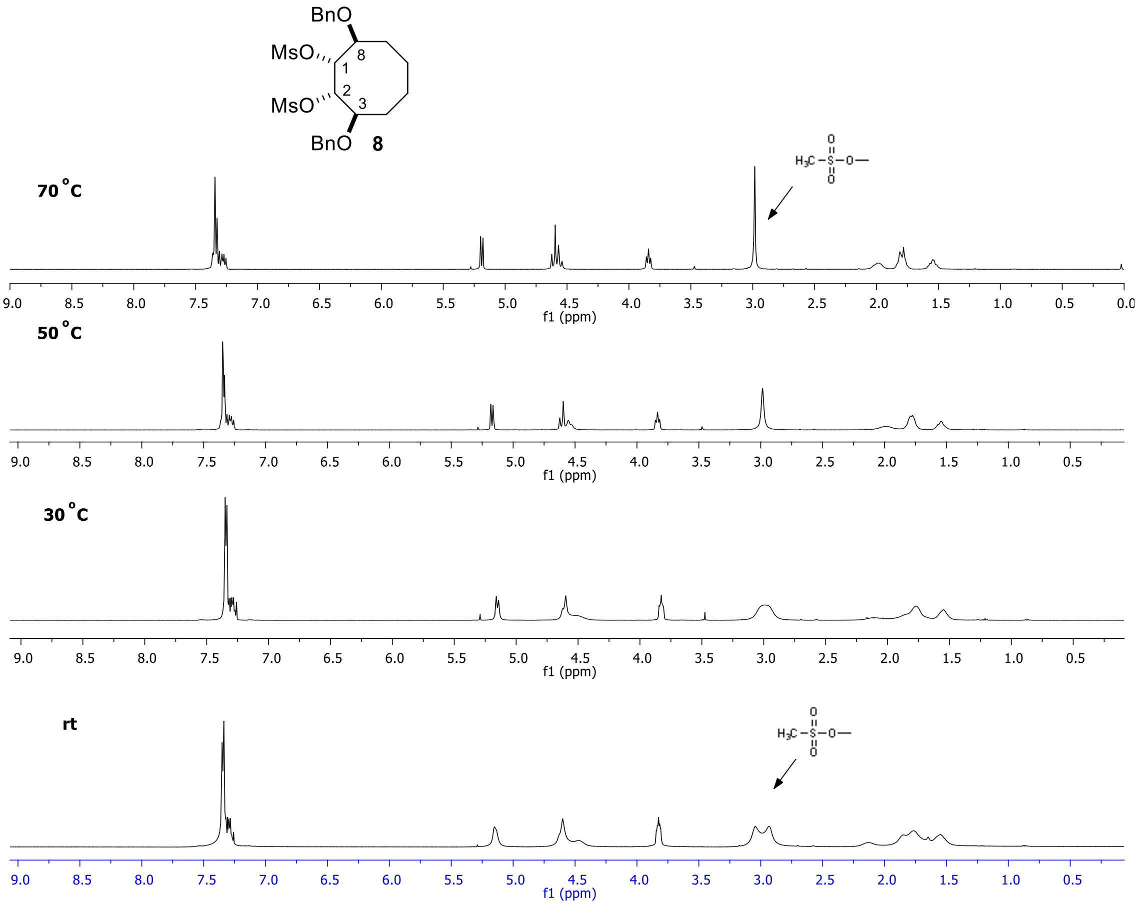

(1*S,2*R**,3*R**,8*S**)-2-Azido-3,8-bis(benzyloxy)cyclooctyl methanesulfonate (10): $CDCl_3$ (^{1}H NMR and ^{13}C NMR)**

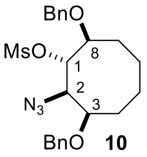

10

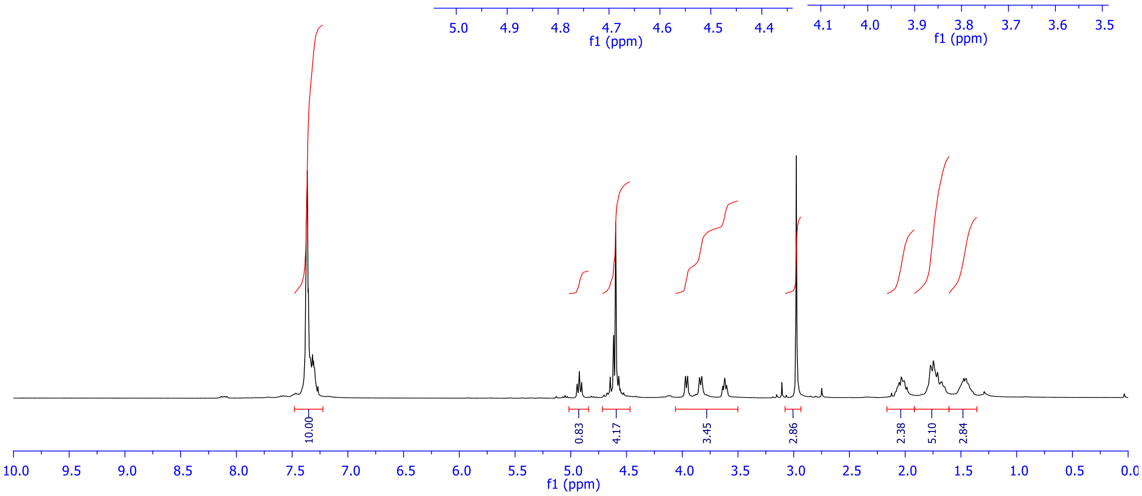

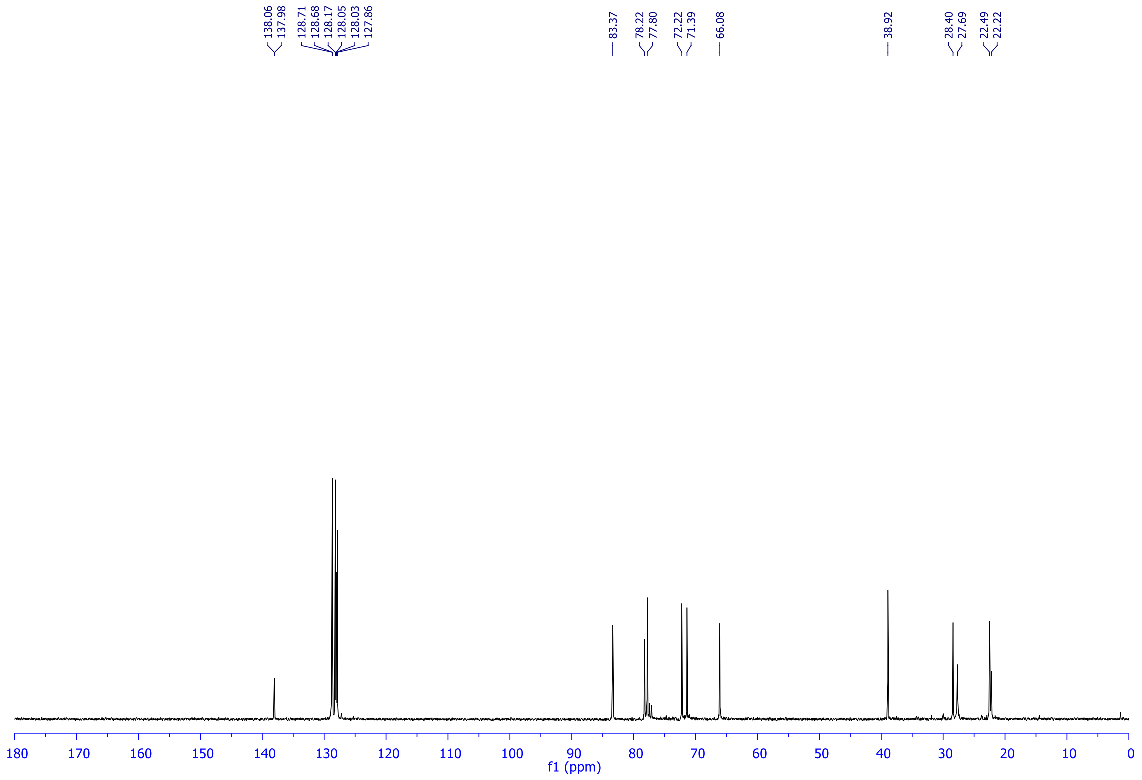

(1*S,2*R**,3*R**,8*S**)-2-Azido-3,8-bis(benzyloxy)cyclooctyl methanesulfonate (10): CDCl$_3$-HMQC**

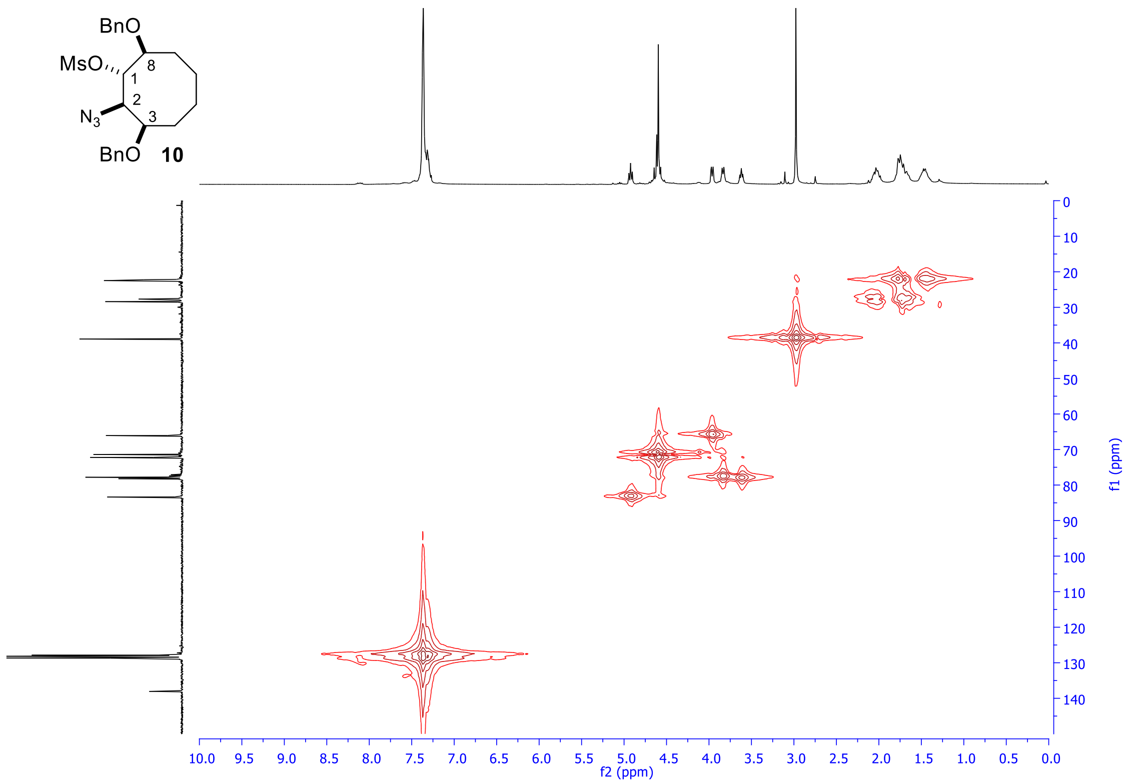

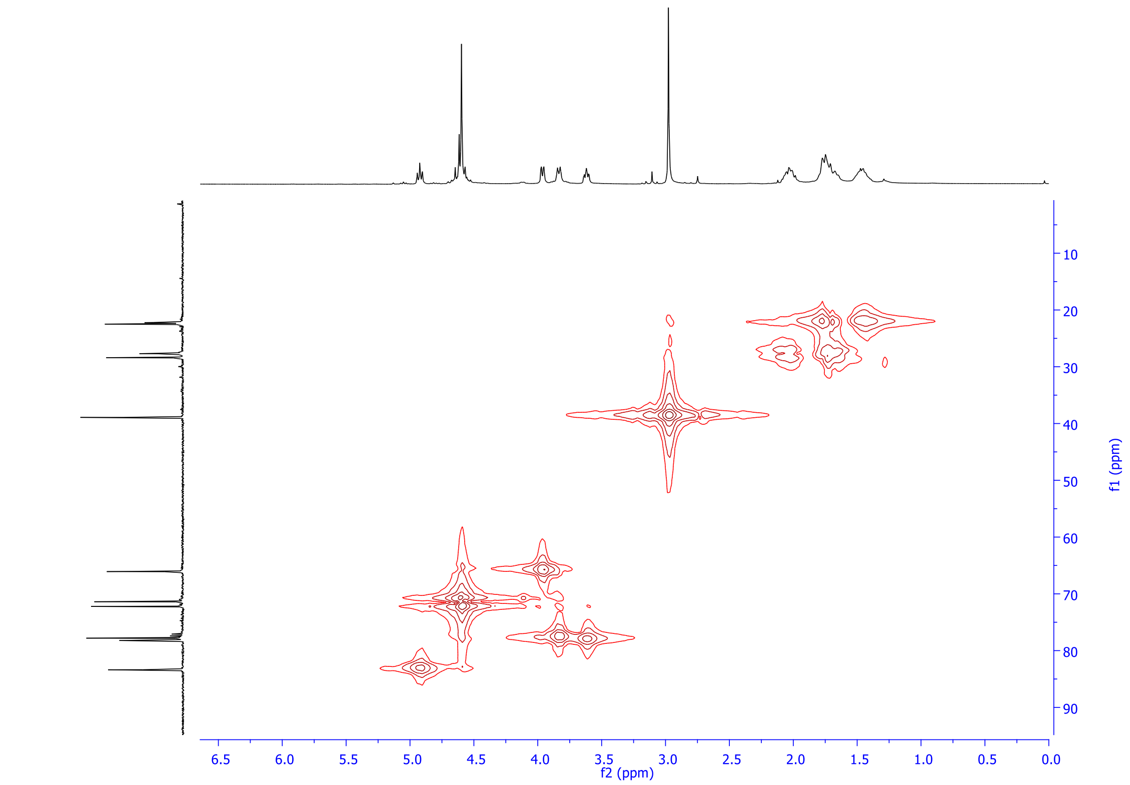

(1*S,2*R**,3*R**,8*S**)-2-Azido-3,8-bis(benzyloxy)cyclooctyl methanesulfonate (10): $CDCl_3$-COSY**

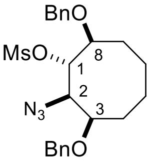

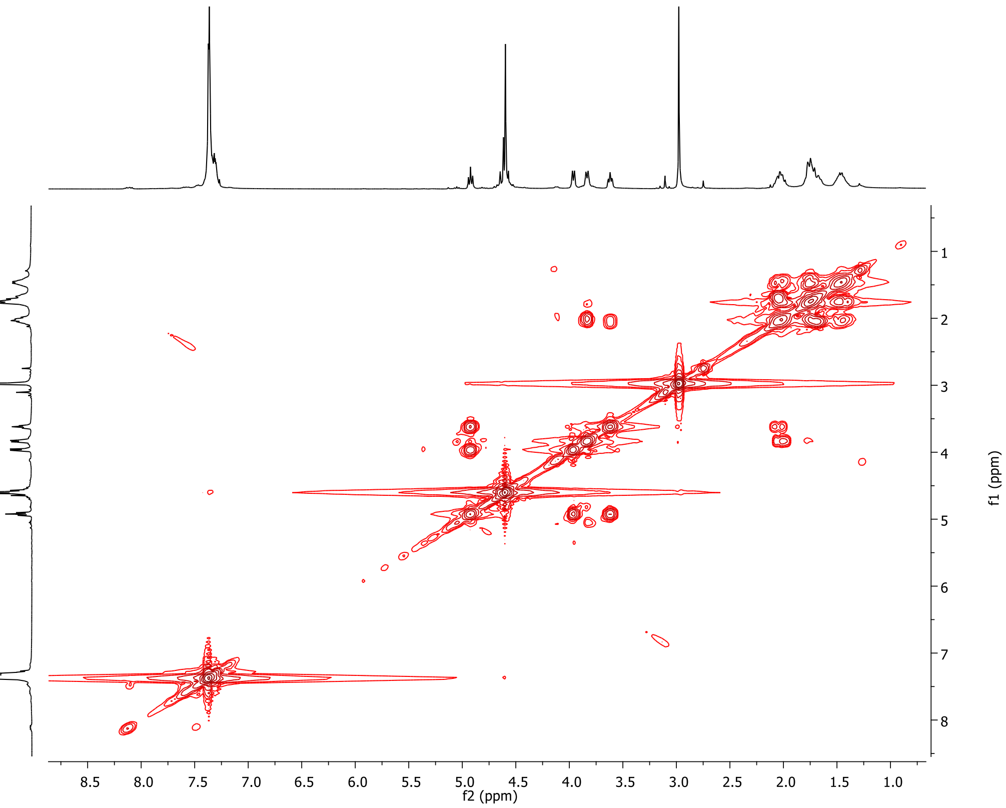

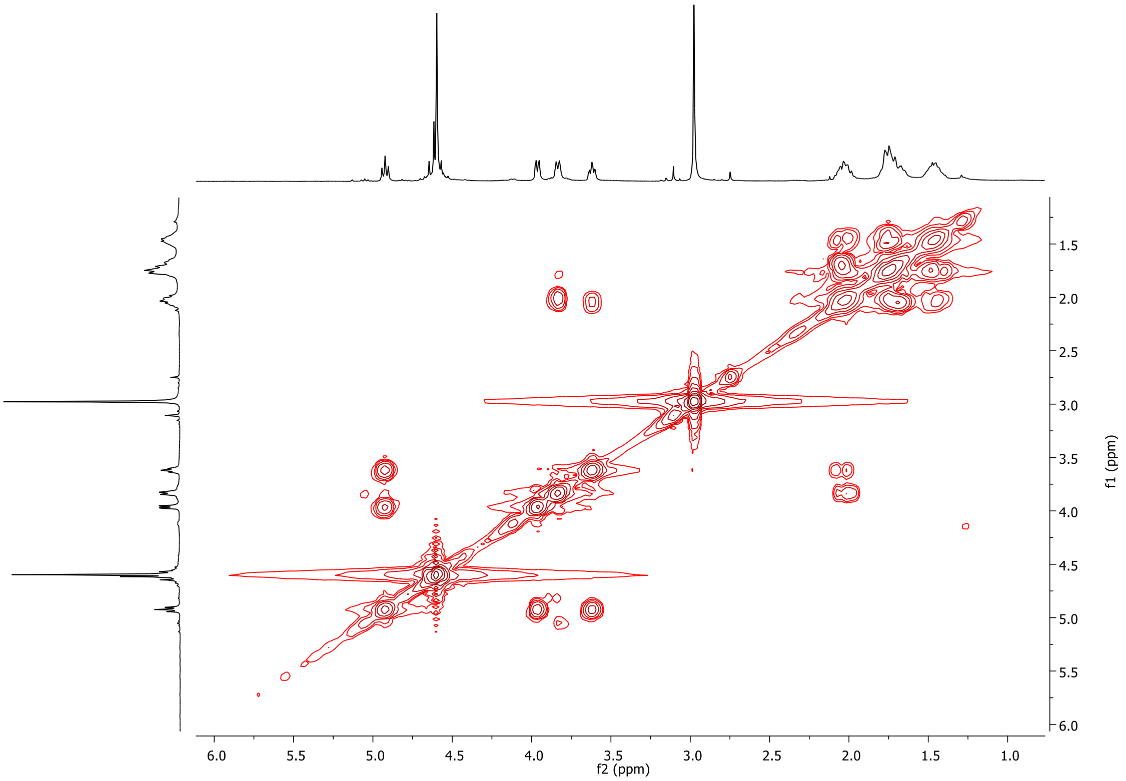

(1*S,2*S**,3*R**,8*S**)-2-Azido-3,8-bis(benzyloxy)cyclooctan-1-ol (11): CDCl₃ (¹H NMR and ¹³C NMR)**

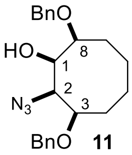

11

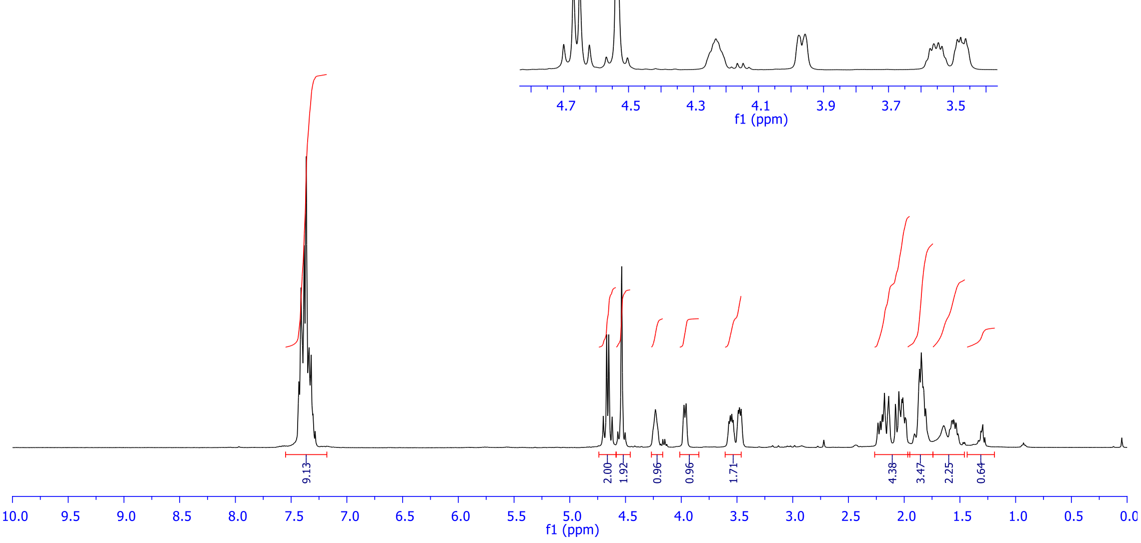

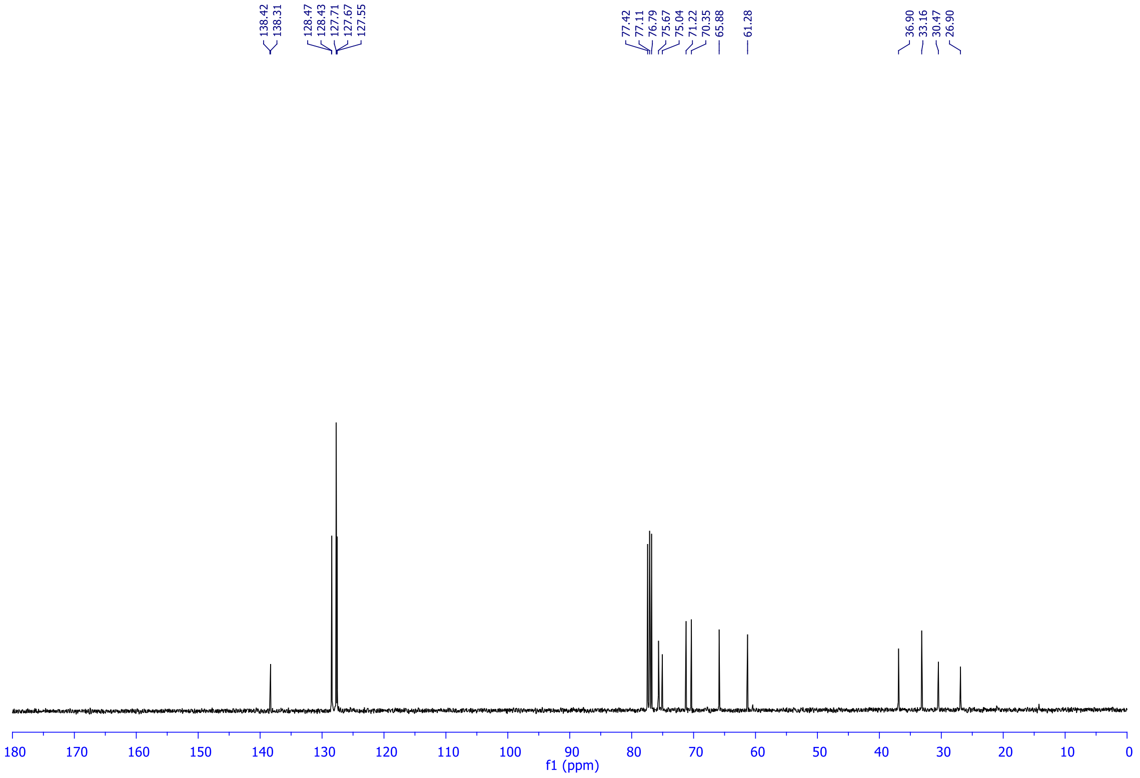

(1*S,2*S**,3*R**,8*S**)-2-Azido-3,8-bis(benzyloxy)cyclooctan-1-ol (11): $CDCl_3$-HMQC**

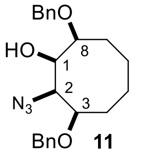

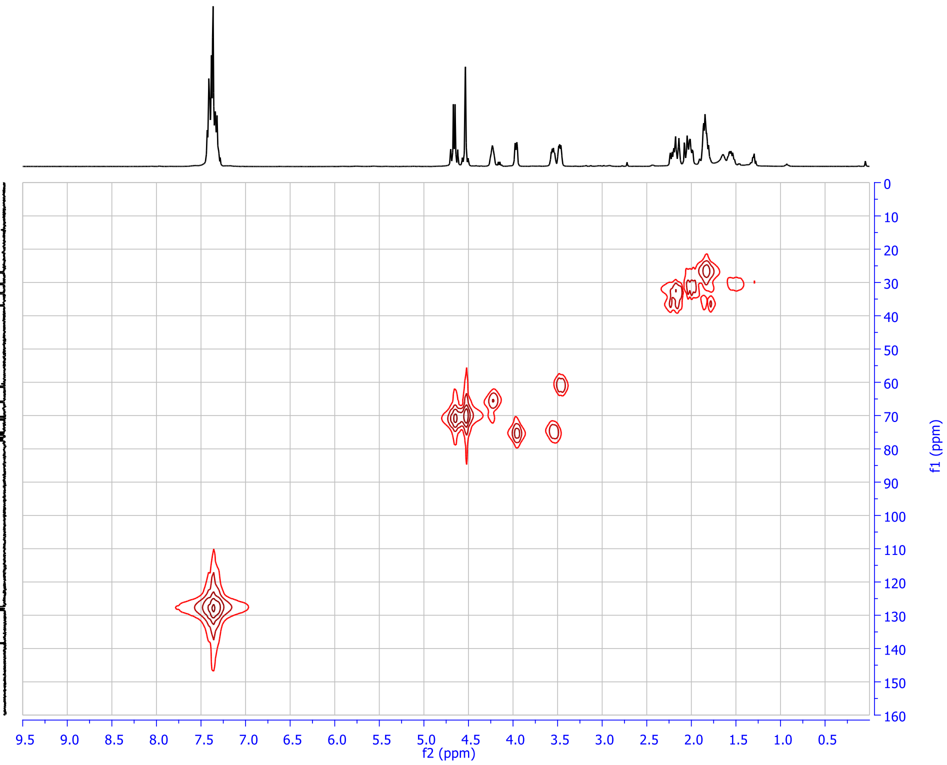

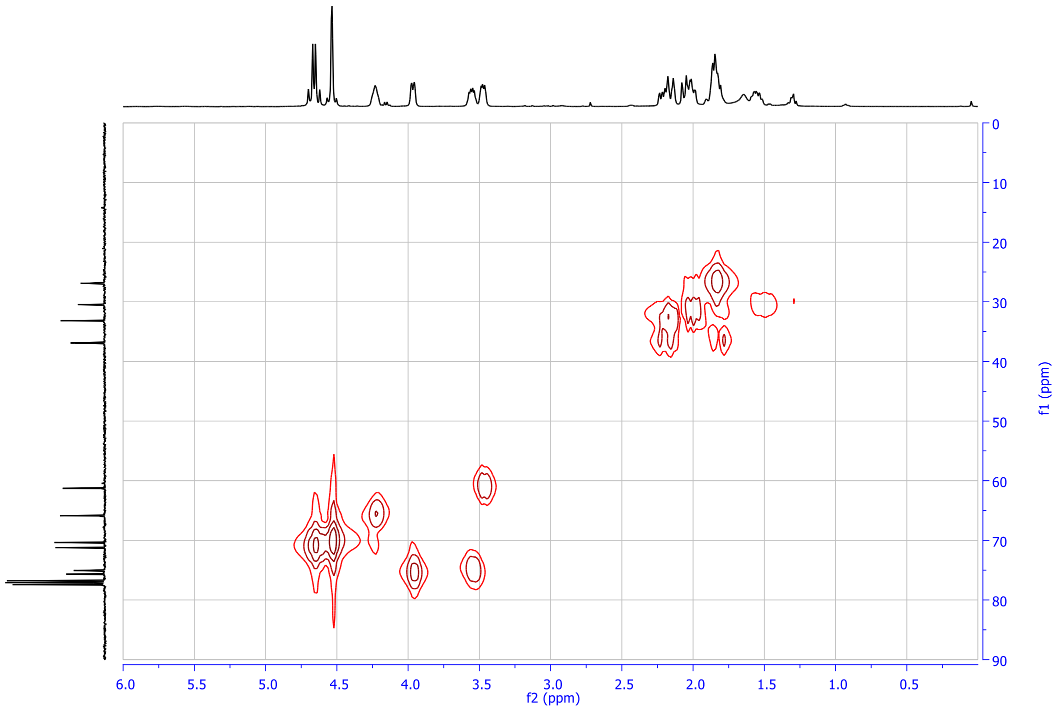

(1*S,2*S**,3*R**,8*S**)-2-Azido-3,8-bis(benzyloxy)cyclooctan-1-ol (11): $CDCl_3$-COSY**

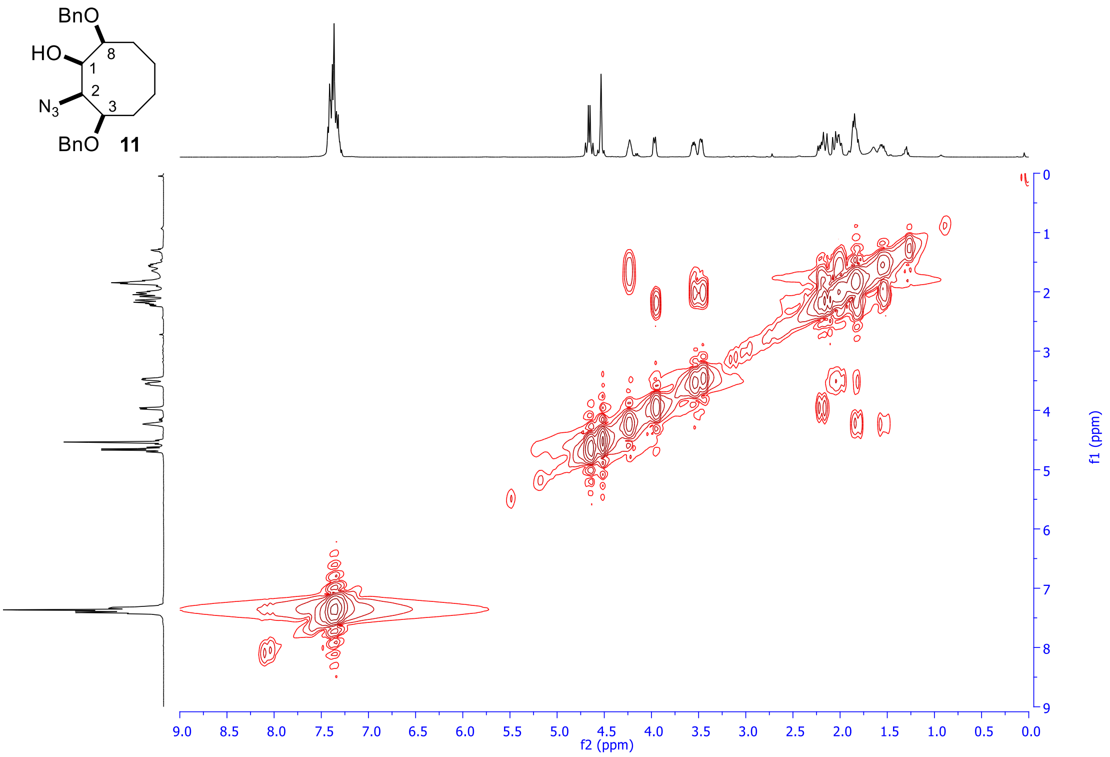

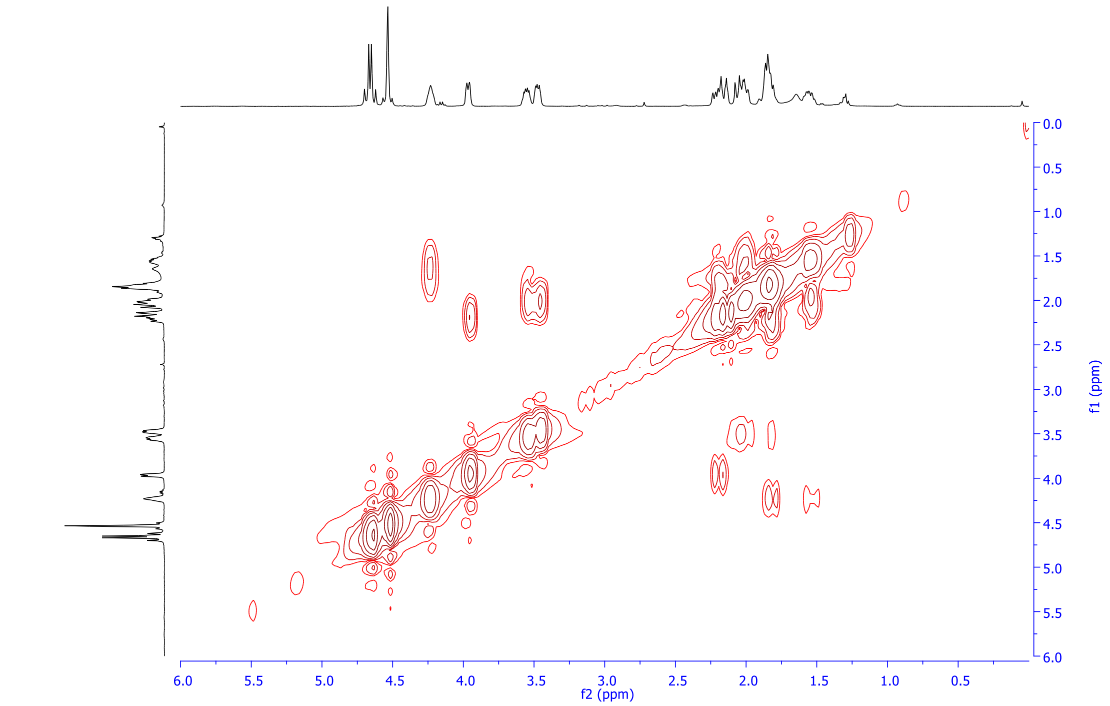

(1*S,2*S**,3*R**,8*S**)-2-Azido-3,8-bis(benzyloxy)cyclooctan-1-ol (11): NOE-Dif spectra**

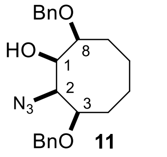

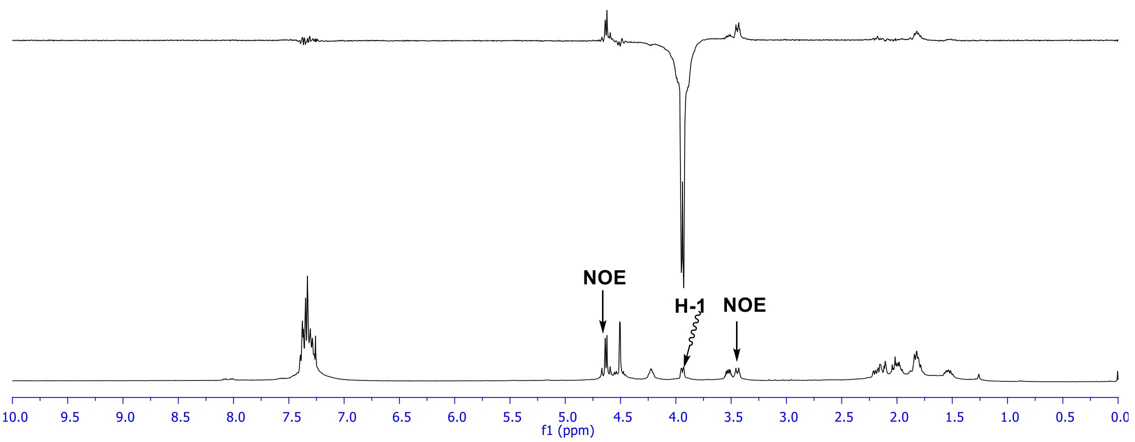

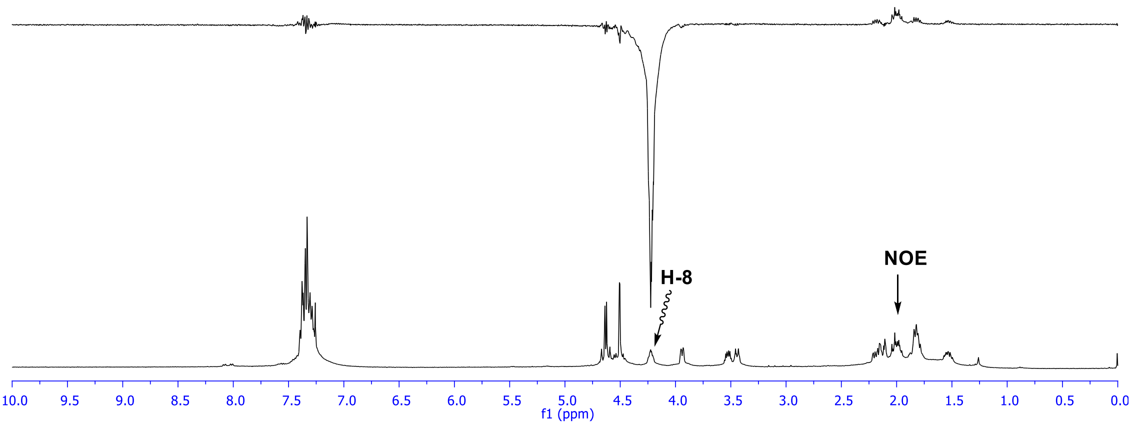

(1*R,2*S**,3*R**,8*S**)-2-Amino-3,8-bis(benzyloxy)cyclooctan-1-ol (12): $CDCl_3$ (^{1}H NMR and ^{13}C NMR)**

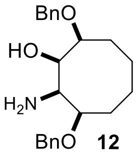

12

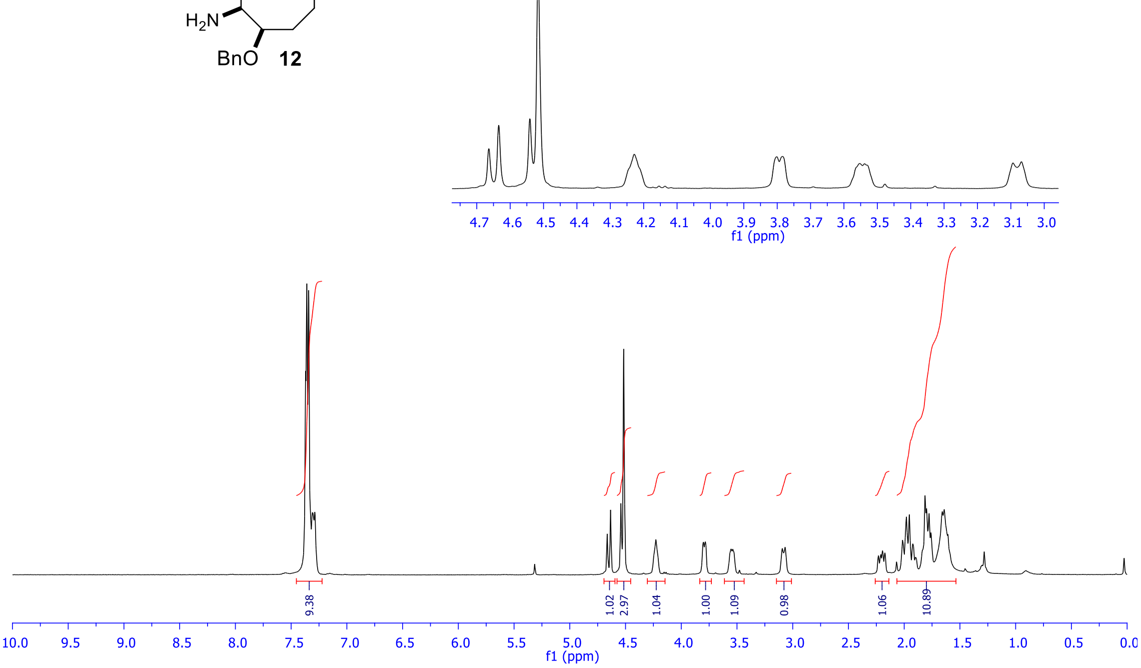

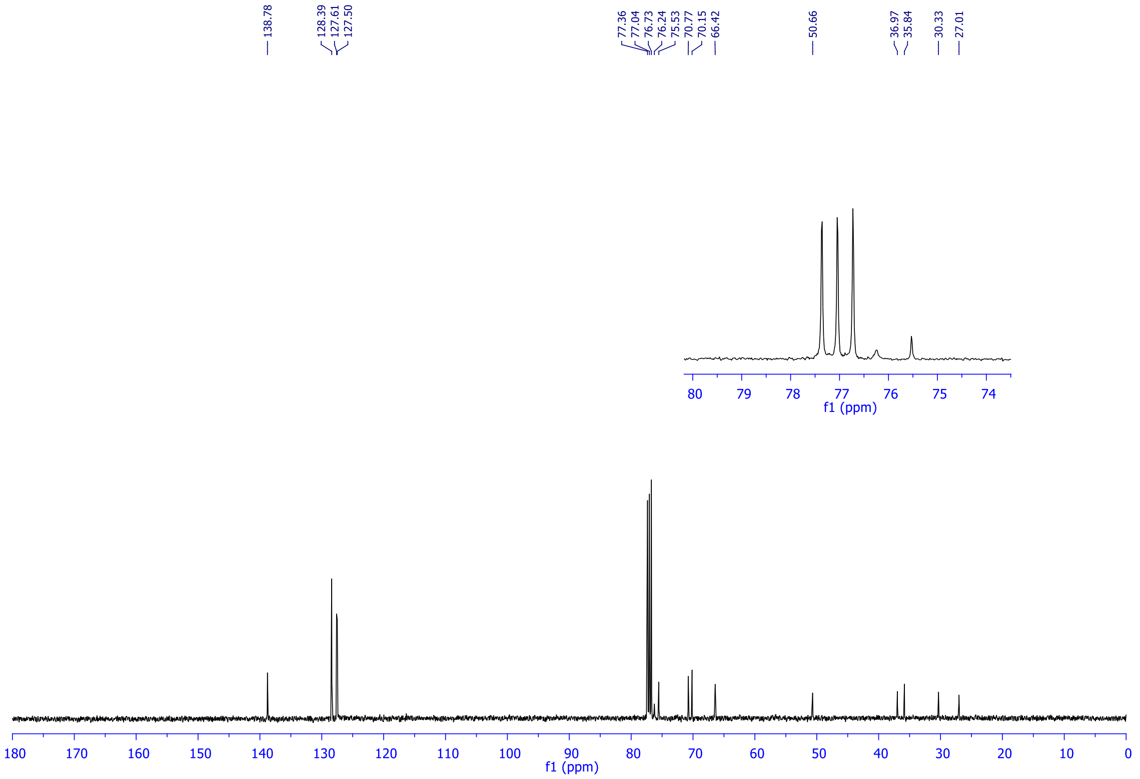

(1*R,2*S**,3*R**,8*S**)-2-Amino-3,8-bis(benzyloxy)cyclooctan-1-ol (12): CDCl₃-HMQC**

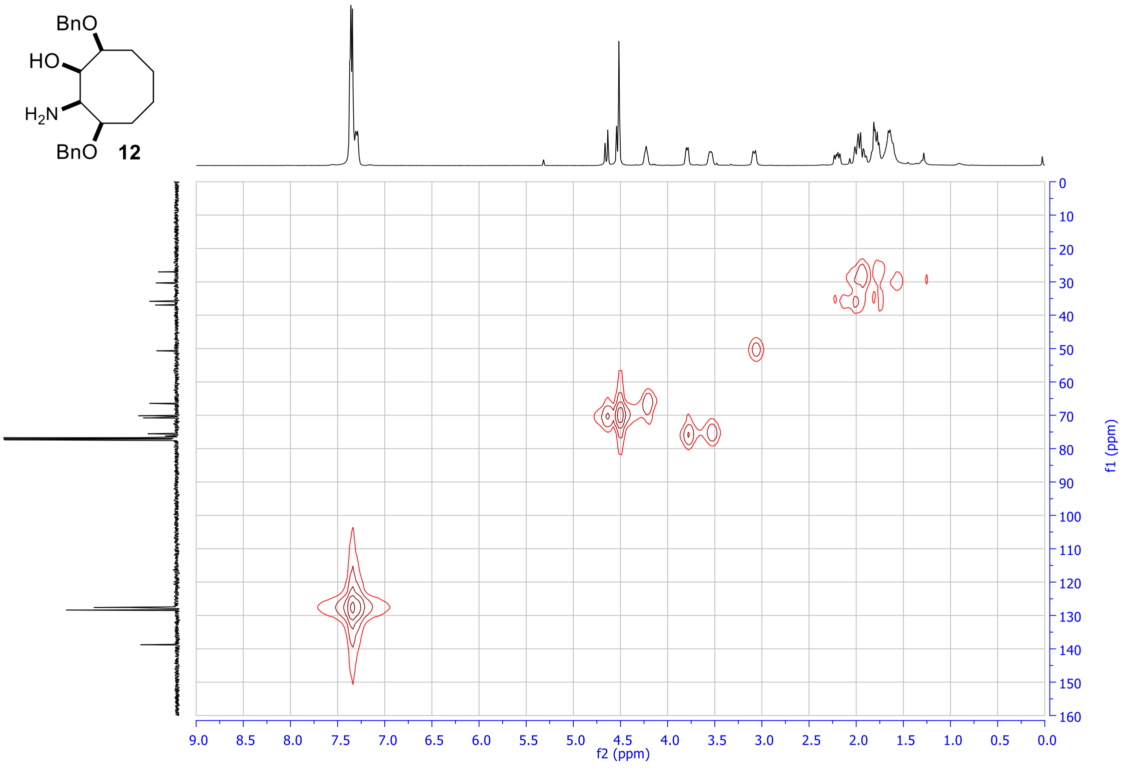

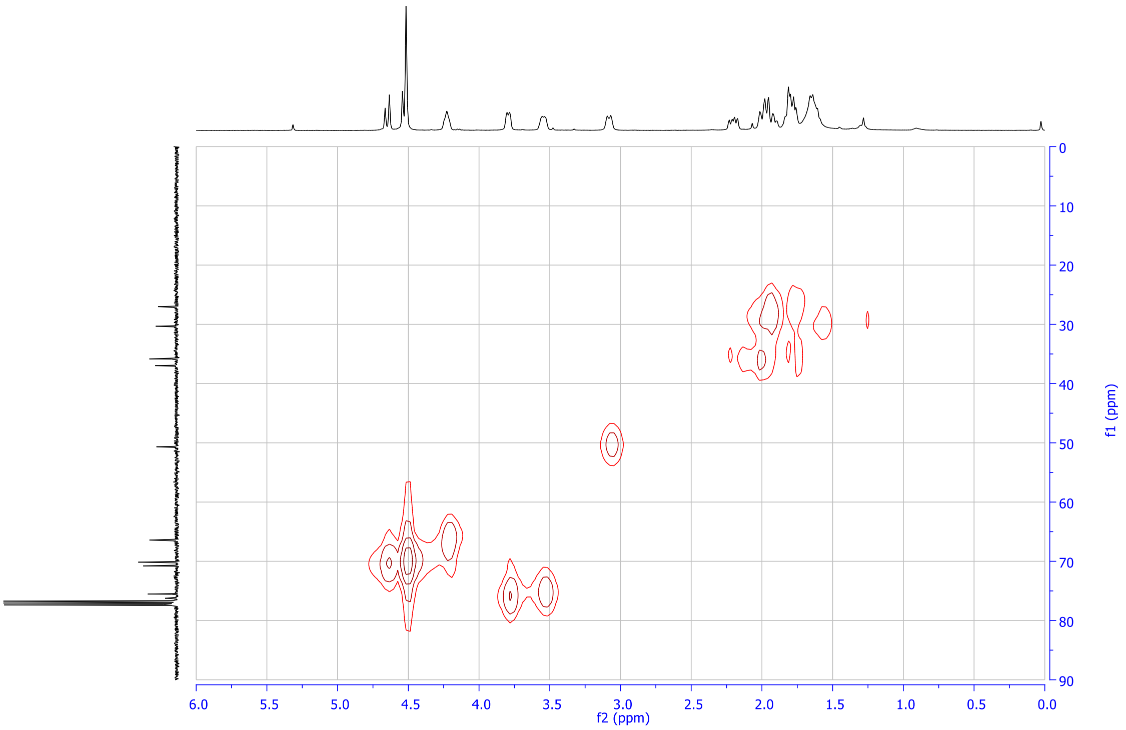

(1*S,2*R**,3*R**,4*R**)-3-Aminocyclooctane-1,2,4-triol (13): CD_3OD (^{1}H NMR and ^{13}C NMR)**

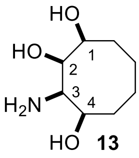

13

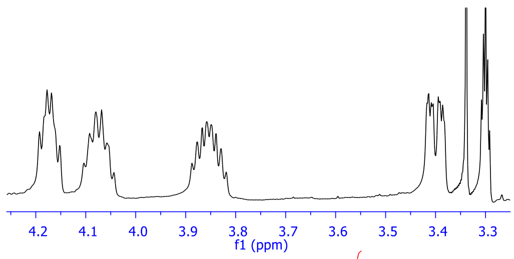

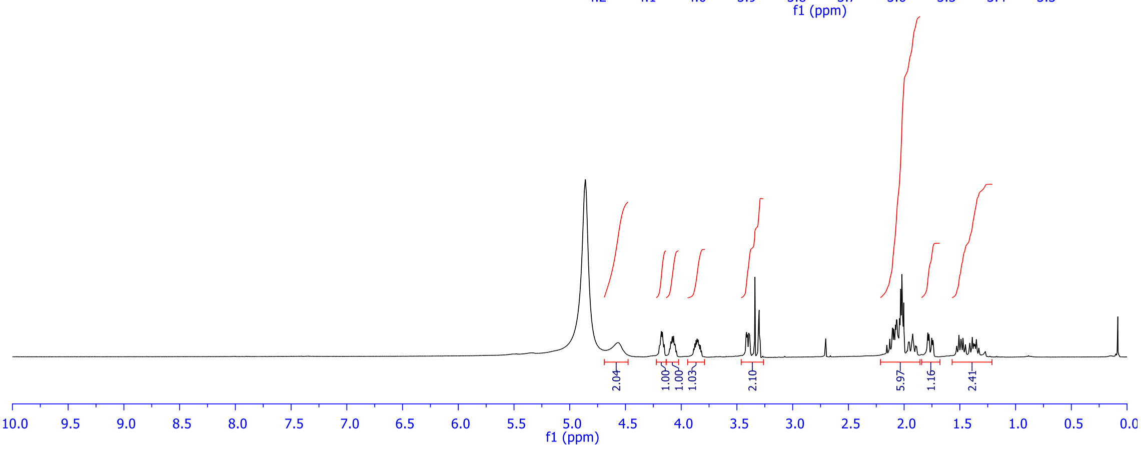

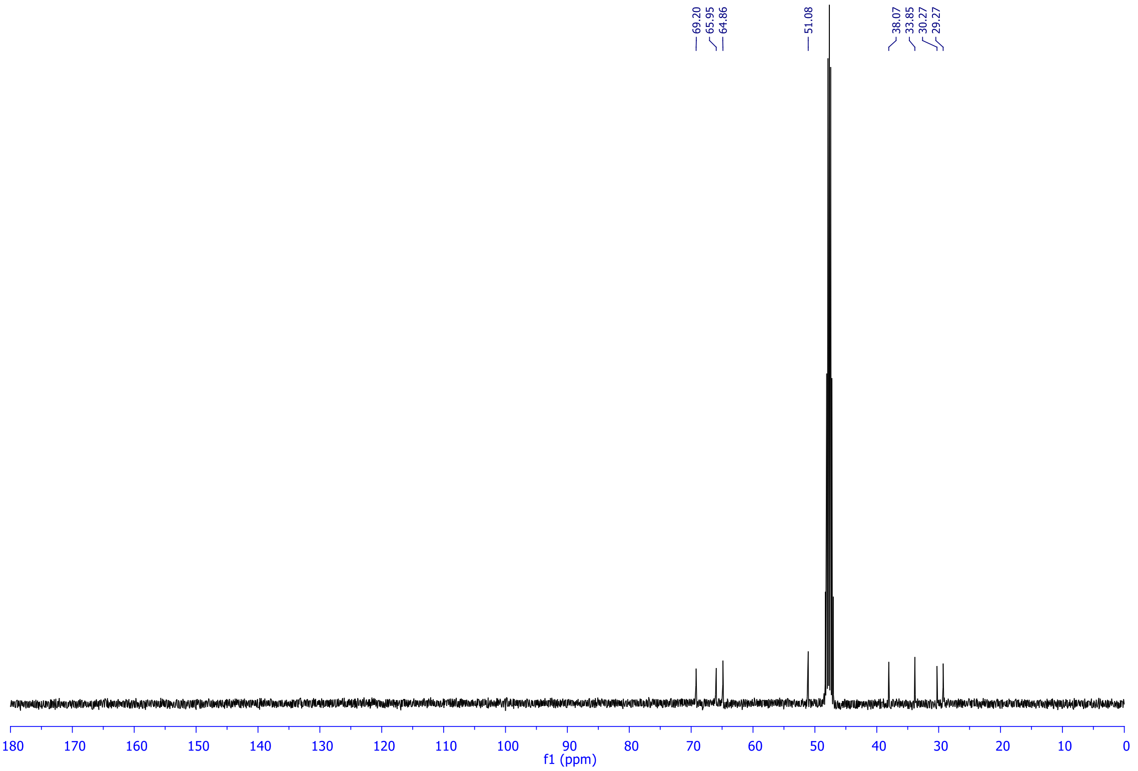

(1*S,2*R**,3*R**,4*R**)-3-Aminocyclooctane-1,2,4-triol (13): CD_3OD (Double Resonances)**

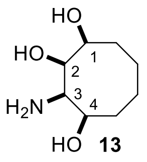

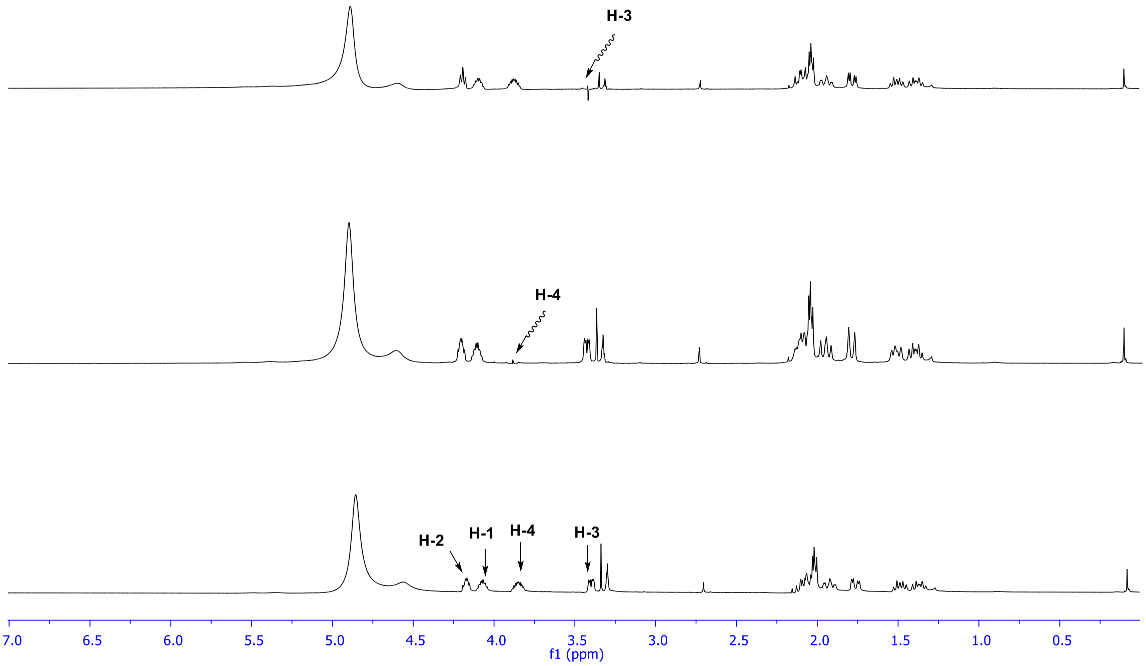

(1*S,2*R**,3*R**,4*R**)-3-Aminocyclooctane-1,2,4-triol (13): CD_3OD-HMQC**

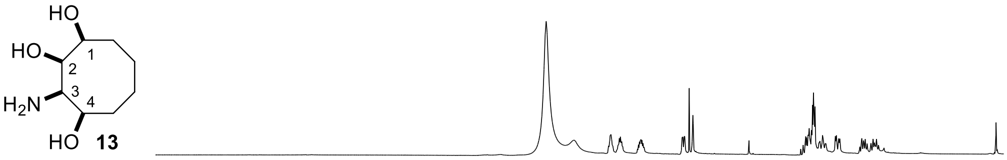

13

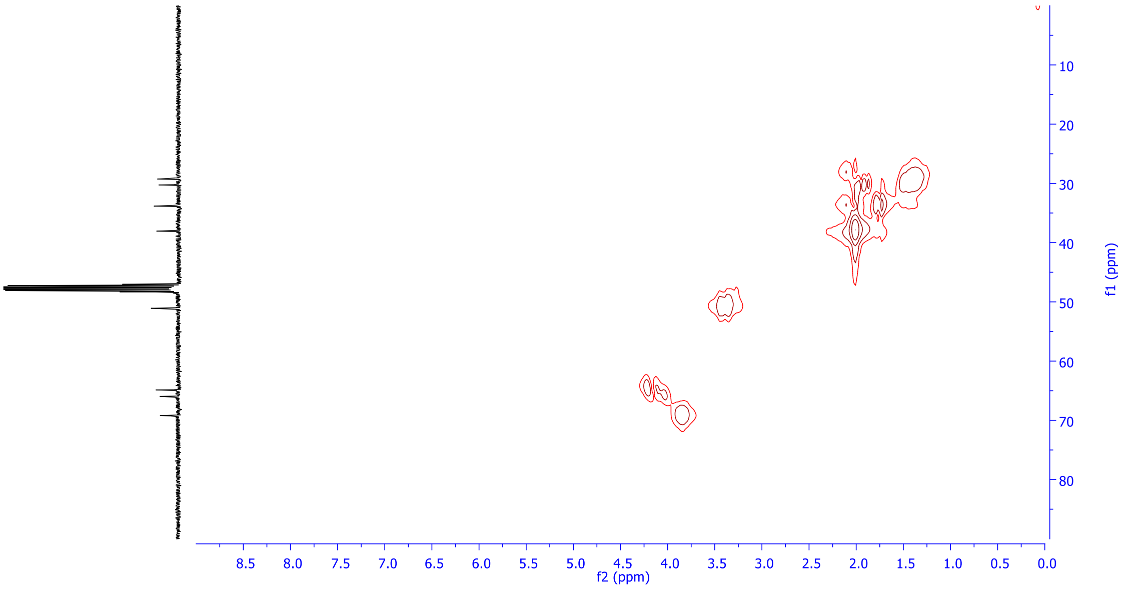

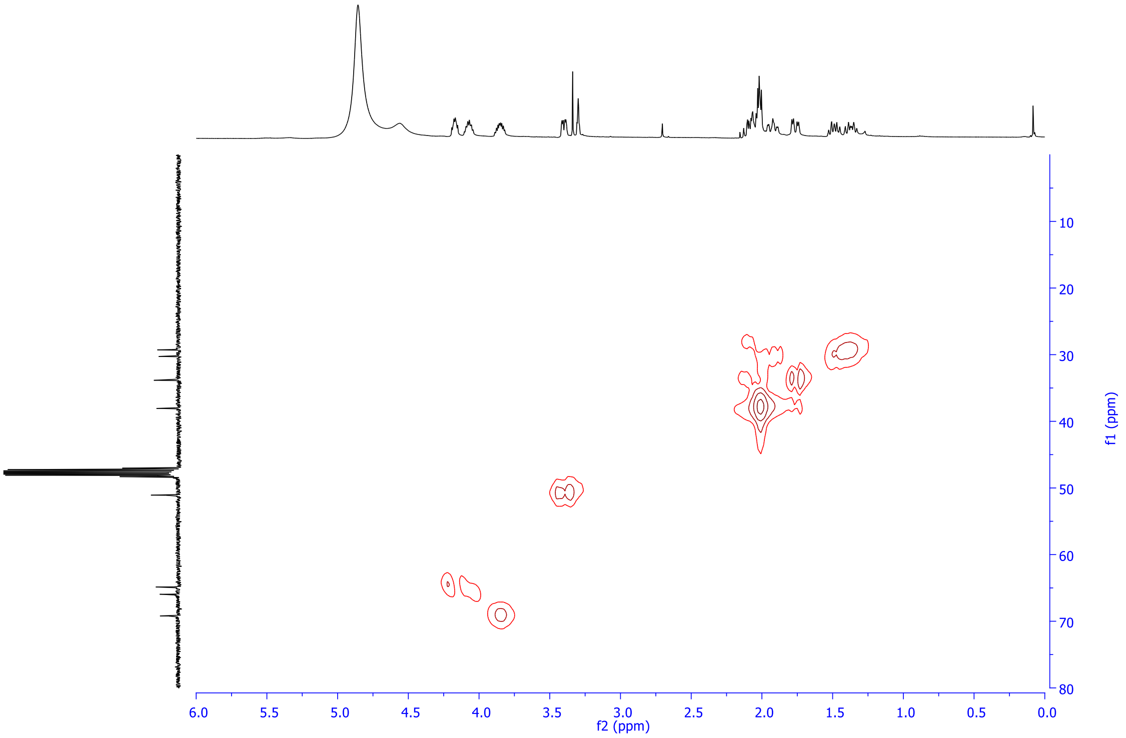

(1*R,2*S**,7*R**,8*S**)-2,7-Bis(benzyloxy)-9-azabicyclo[6.1.0]nonane (14): $CDCl_3$ (^{1}H NMR and ^{13}C NMR)**

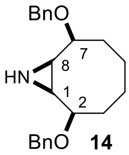

14

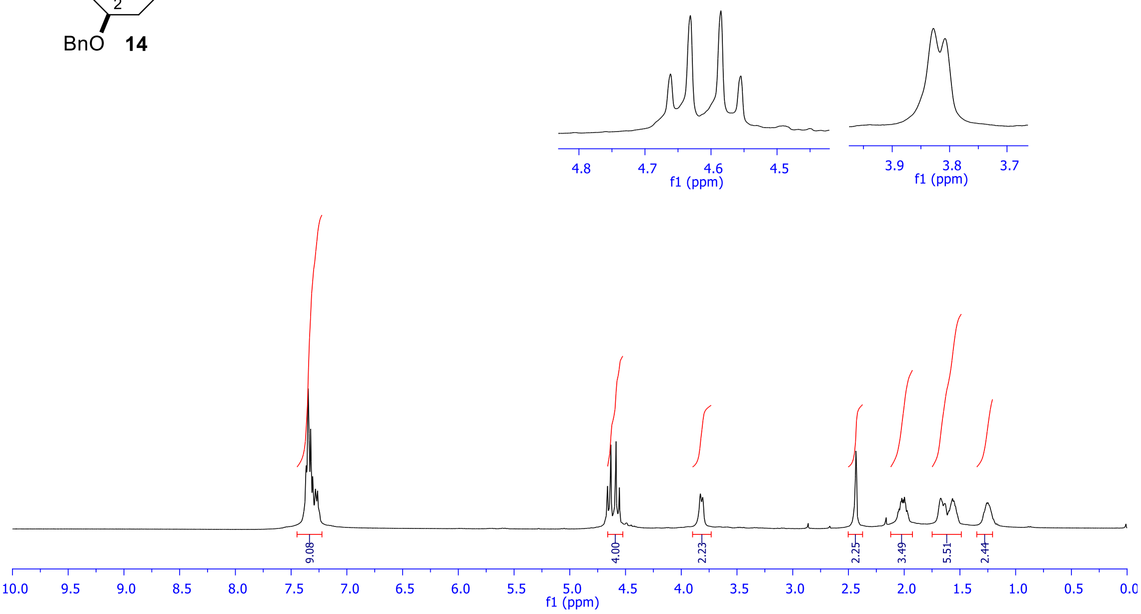

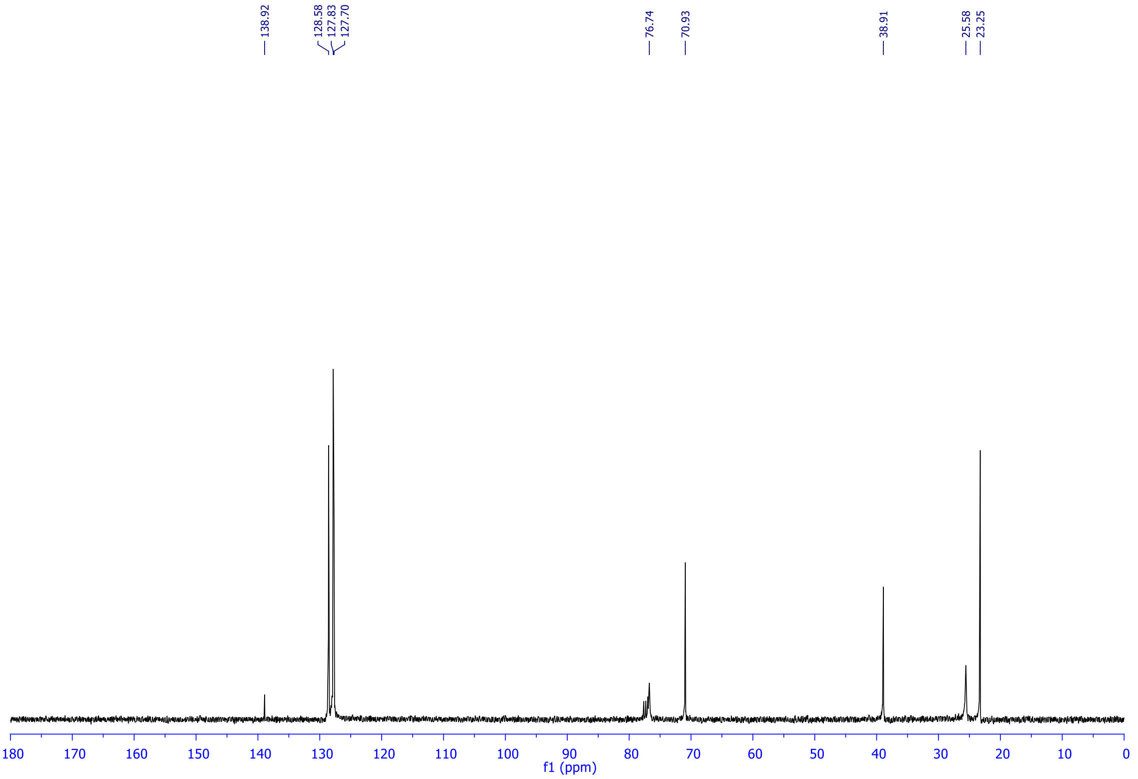

***tert*-Butyl (1*R**,2*S**,7*R**,8*S**)-2,7-bis(benzyloxy)-9-azabicyclo[6.1.0]nonane-9-carboxylate (15): $CDCl_3$ (^{1}H NMR and ^{13}C NMR)**

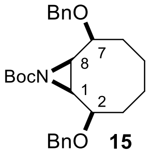

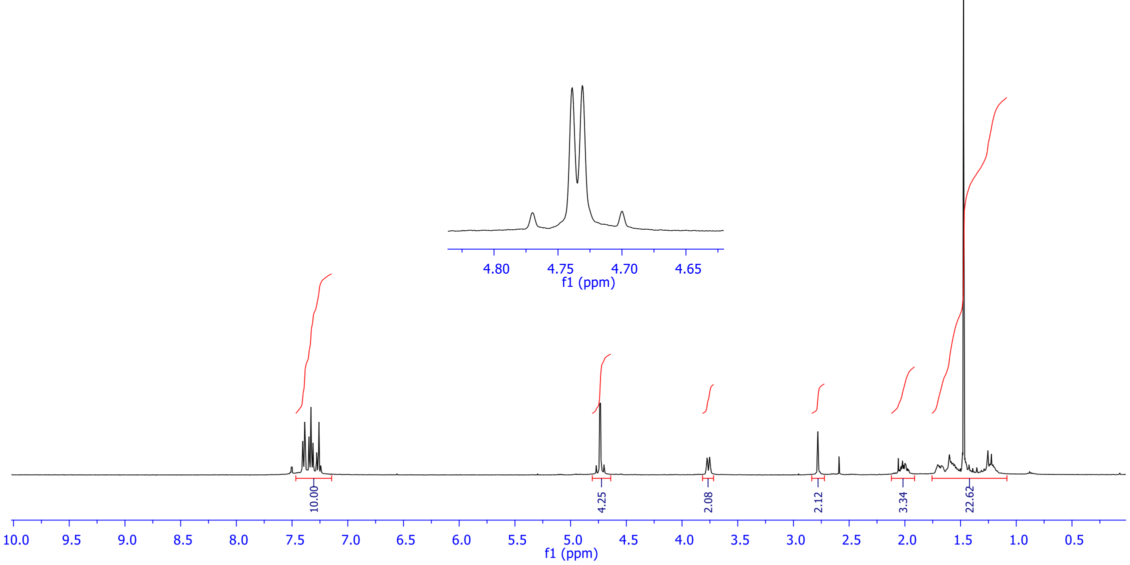

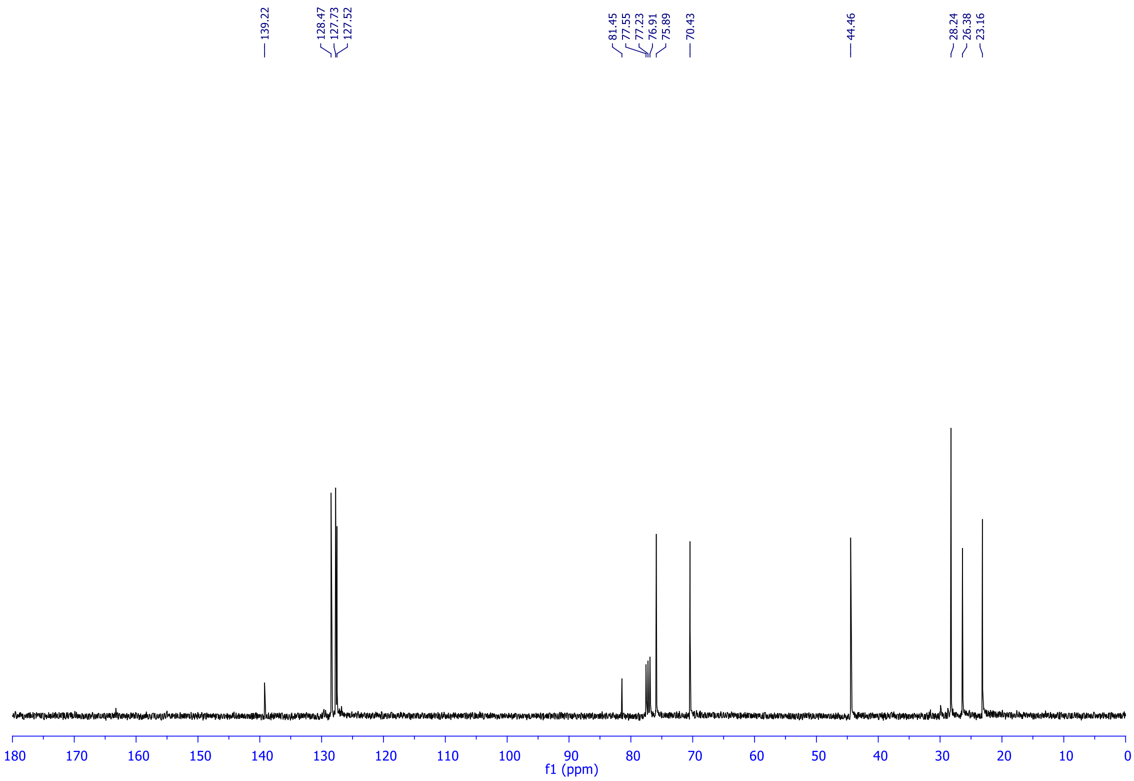

***tert*-Butyl (1*R**,2*S**,7*R**,8*S**)-2,7-bis(benzyloxy)-9-azabicyclo[6.1.0]nonane-9-carboxylate (15): CDCl3-HMQC**

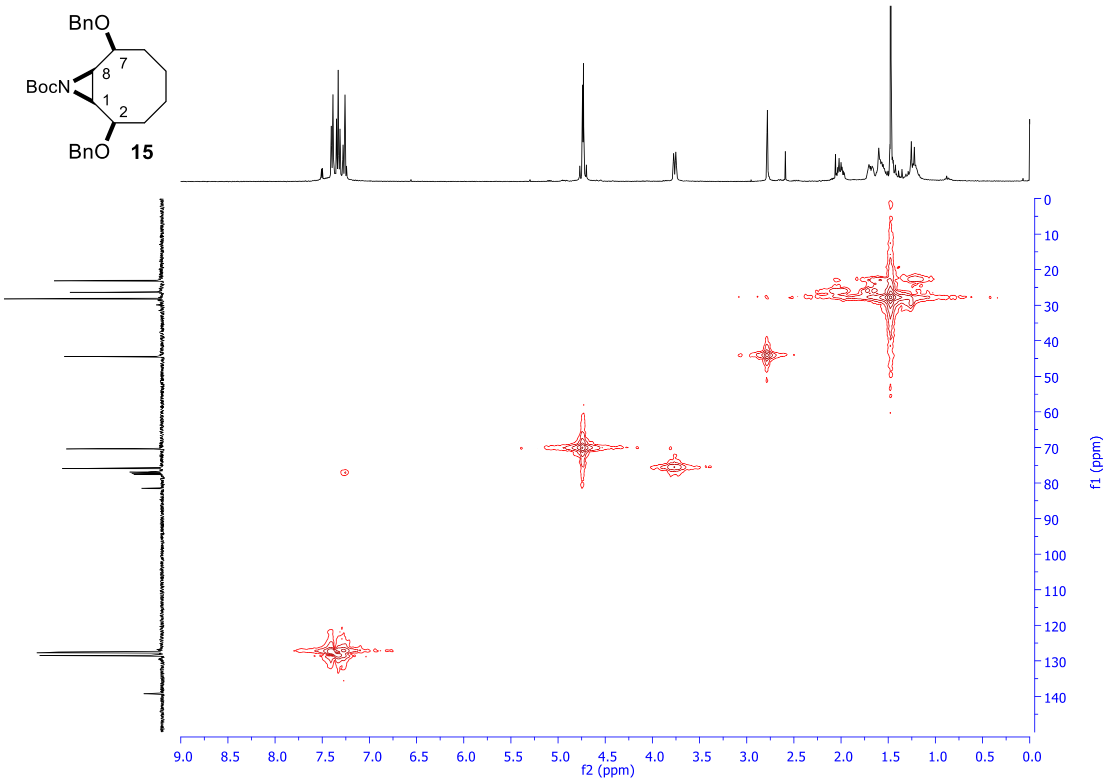

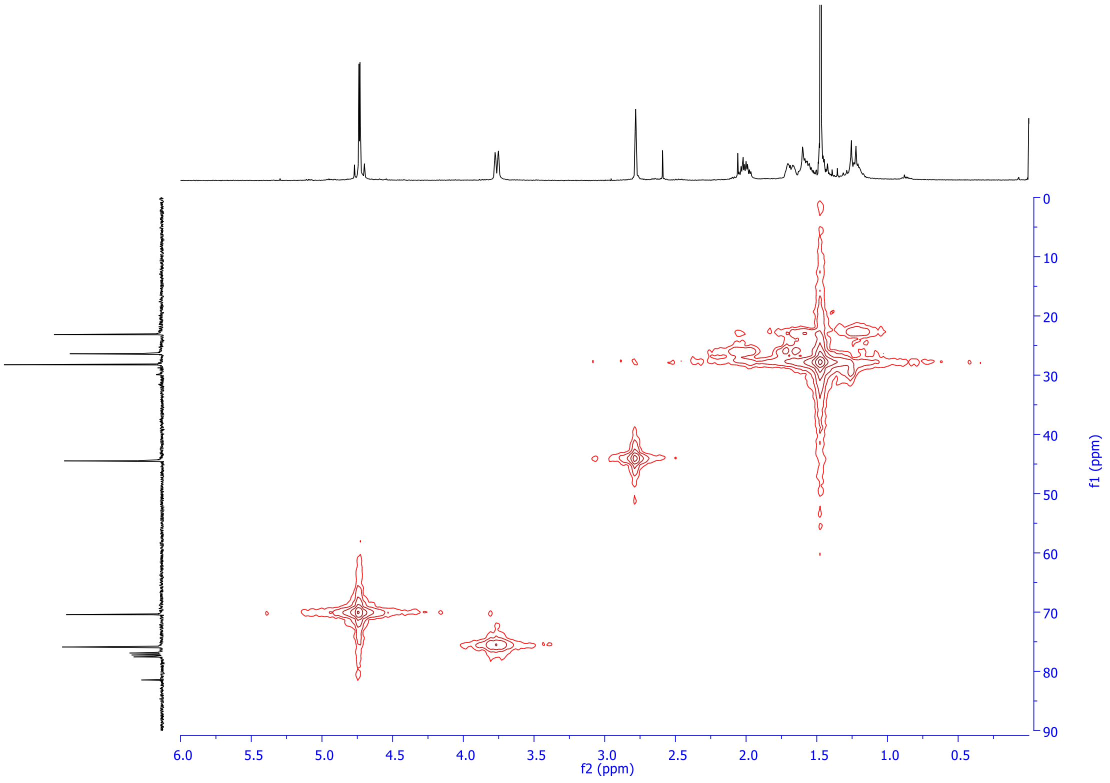

***tert*-Butyl (1*R**,2*S**,7*R**,8*S**)-2,7-bis(benzyloxy)-9-azabicyclo[6.1.0]nonane-9-carboxylate (15): $CDCl_3$-COSY**

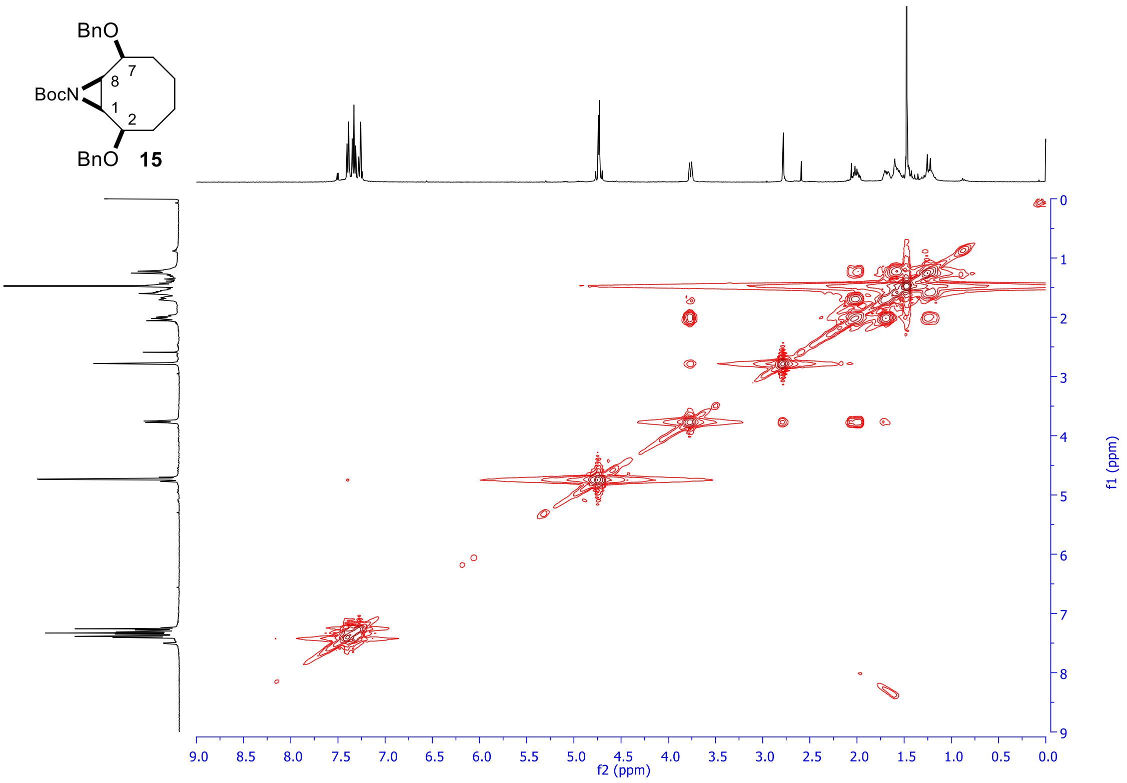

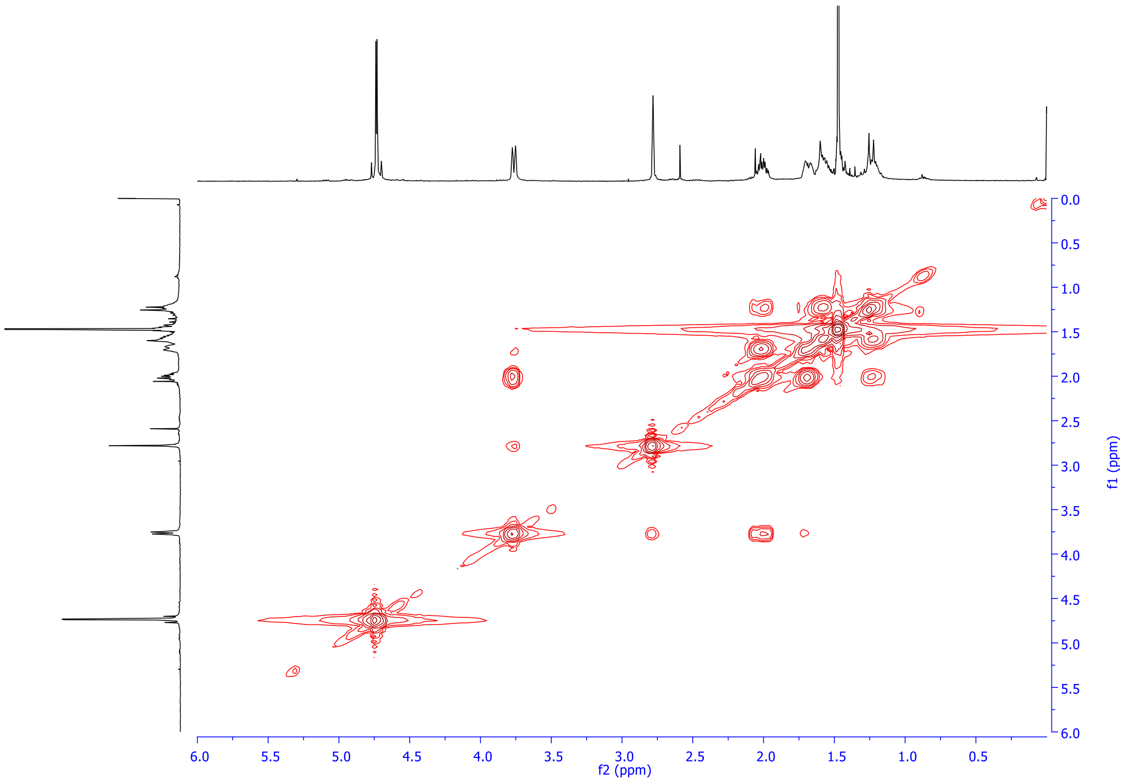

tert-Butyl (1_R_*,2_S_*,7_R_*,8_S_*)-2,7-bis(benzyloxy)-9-azabicyclo[6.1.0]nonane-9-carboxylate (15): NOE-dif spectra

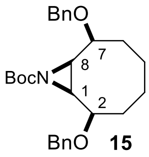

15

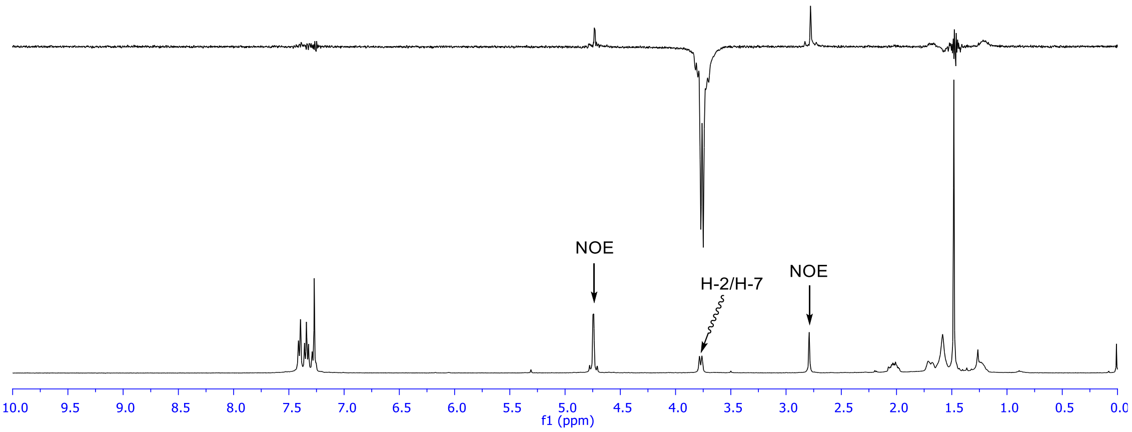

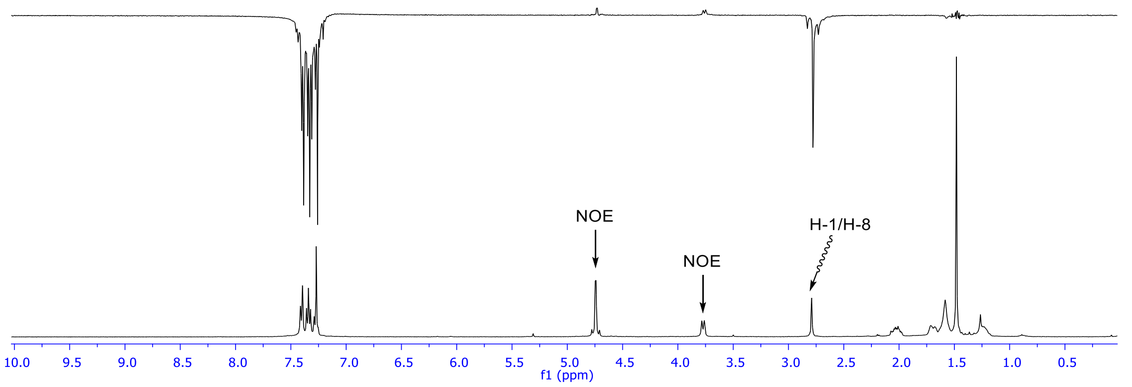

(1*R,2*S**,7*R**,8*S**)-9-Azabicyclo[6.1.0]nonane-2,7-diol (16): D_2O (^1H NMR and ^{13}C NMR)**

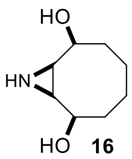

16

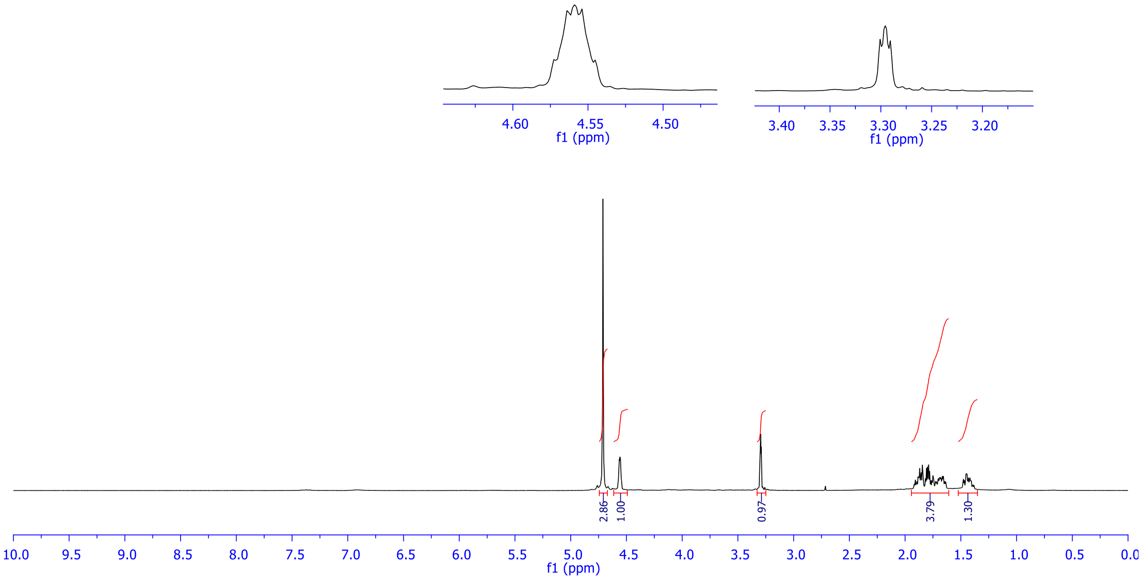

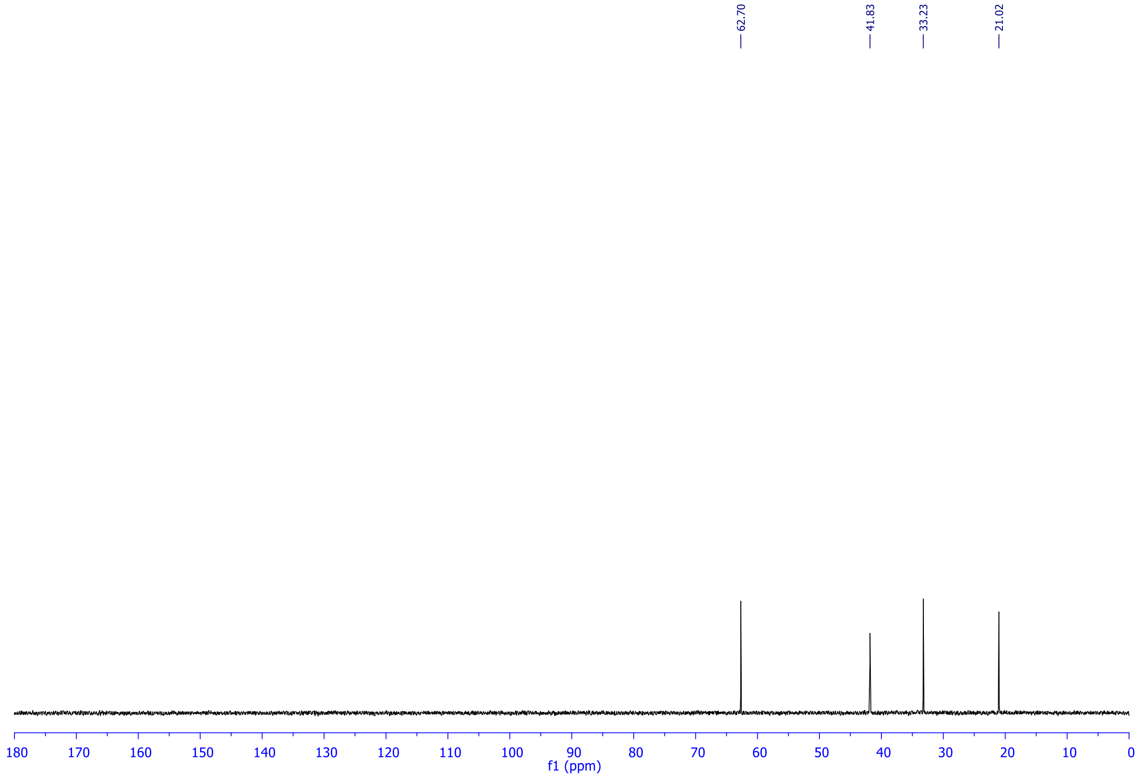

Supplement: File 1 — Experimental section, 1H and 13C NMR spectra for all new compounds, as well as selected 2D NMR spectra are provided. [file Beilstein_J_Org_Chem-18-1539-s001.pdf]
